# Neural Correlates of Verbal Working Memory: An fMRI Meta-Analysis

**DOI:** 10.3389/fnhum.2019.00180

**Published:** 2019-06-12

**Authors:** Mónica Emch, Claudia C. von Bastian, Kathrin Koch

**Affiliations:** ^1^Department of Neuroradiology, School of Medicine, Klinikum Rechts der Isar, Technical University of Munich, Munich, Germany; ^2^TUM-Neuroimaging Center (TUM-NIC), Technical University of Munich, Munich, Germany; ^3^Graduate School of Systemic Neurosciences, Ludwig-Maximilians-Universität, Martinsried, Germany; ^4^Department of Psychology, University of Sheffield, Sheffield, United Kingdom

**Keywords:** verbal working memory, meta-analysis, neuroimaging, fMRI, subcortical areas, fronto-parietal activation, right cerebellum

## Abstract

Verbal Working memory (vWM) capacity measures the ability to maintain and manipulate verbal information for a short period of time. The specific neural correlates of this construct are still a matter of debate. The aim of this study was to conduct a coordinate-based meta-analysis of 42 fMRI studies on visual vWM in healthy subjects (*n* = 795, males = 459, females = 325, unknown = 11; age range: 18–75). The studies were obtained after an exhaustive literature search on PubMed, Scopus, Web of Science, and Brainmap database. We analyzed regional activation differences during fMRI tasks with the anisotropic effect-size version of seed-based d mapping software (ES-SDM). The results were further validated by performing jackknife sensitivity analyses and heterogeneity analyses. We investigated the effect of numerous relevant influencing factors by fitting corresponding linear regression models. We isolated consistent activation in a network containing fronto-parietal areas, right cerebellum, and basal ganglia structures. Regarding lateralization, the results pointed toward a bilateral frontal activation, a left-lateralization of parietal regions and a right-lateralization of the cerebellum, indicating that the left-hemisphere concept of vWM should be reconsidered. We also isolated activation in regions important for response inhibition, emphasizing the role of attentional control in vWM. Moreover, we found a significant influence of mean reaction time, load, and age on activation associated with vWM. Activation in left medial frontal gyrus, left precentral gyrus, and left precentral gyrus turned out to be positively associated with mean reaction time whereas load was associated with activation across the PFC, fusiform gyrus, parietal cortex, and parts of the cerebellum. In the latter case activation was mainly detectable in both hemispheres whereas the influence of age became manifest predominantly in the left hemisphere. This led us to conclude that future vWM studies should take these factors into consideration.

## Introduction

Working memory (WM) is a cognitive system that holds information available that is needed for complex cognition in the present moment (Baddeley, 2010; Oberauer and Hein, 2012). It has been shown that WM capacity is a strong predictor of a wide range of complex cognitive tasks such as analytic problem solving, language acquisition, and reading comprehension (Daneman and Carpenter, 1980; Engle et al., 1999; Wiley and Jarosz, 2012). There have been several attempts to understand the organization of human WM. The arguably most influential model is the multiple-component model proposed by Baddeley and Hitch (1974). The authors hypothesized the existence of a “central executive” component, which controls the incoming information and passes the information to two subsystems: the “phonological loop” and the “visuospatial sketchpad.” Within the phonological loop, due to the interplay of its two components—the phonological store and the articulatory loop—the verbal material representation can be kept in an active state. Verbal information is processed in perceptual systems before it enters the phonological loop in which it is temporarily stored in the phonological store and maintained through the articulatory loop using subvocal rehearsal of the information. In addition to subvocal rehearsal, the articulatory loop is also thought to be involved whenever verbal information is presented visually: whereas auditory verbal information (e.g., spoken words) can directly enter the phonological store, visually presented verbal information (e.g., written words) must first be recoded into phonological information. In other words, subvocalization is necessary in order to reroute visually derived verbal material into the phonological store (Buchsbaum and D'Esposito, 2008). The visuospatial sketchpad is responsible for integrating visual and spatial information. Later, the “episodic buffer” was added (Baddeley, 2000). It binds the information from the different subsystems into integrated episodes. Alternative models proposed that WM holds any type of information in a state of heightened availability (Oberauer, 2010; Cowan et al., 2012) whereas others models have emphasized on the role of attentional control in WM (e.g., Kane and Engle, 2003; Unsworth and Engle, 2007). These different theoretical conceptualizations of WM are not necessarily mutually exclusive (Cowan et al., 2012), with common features including a variety of processes such as encoding, maintaining and retrieving information of various domains (e.g., letters, geometric forms, or words), and some attentional control mechanism that supports dealing with interference from irrelevant or distracting information. Thus, the neural correlates of WM may vary depending on the processes, the type of information, and the modality of stimulation (auditory or visual). Given the variety across studies with regard to WM domain and the lack of process differentiation in most studies, the present meta-analysis focused exclusively on visually presented verbal working memory (vWM) across all processes involved in WM.

### Visual Verbal Working Memory

Several fMRI studies over the past years have specifically investigated the brain areas involved in vWM (Honey et al., 2000; Veltman et al., 2003; Chen and Desmond, 2005; Narayanan et al., 2005; Wolf et al., 2006). They basically corroborated the general notion that a variety of brain networks are activated during vWM mainly including areas in the prefrontal cortex (PFC) and the parietal cortex as well as cerebellar and basal ganglia regions (Paulesu et al., 1993; Petrides et al., 1993; Desmond et al., 1997; Crosson et al., 1999; Lewis et al., 2004; Chang et al., 2007; Buchsbaum et al., 2011; Thürling et al., 2012; Moore et al., 2013; Chai et al., 2018). Previous meta-analyses have indicated that the left PFC might be predominantly involved in vWM processes whereas the right PFC seems to be more strongly involved in spatial WM, leading to a lateralization of this region due to different input (Wager and Smith, 2003; Owen et al., 2005). However, there is no general consensus on the functional organization of the PFC (Eriksson et al., 2015). Functional neuroimaging studies suggested that the articulatory loop is associated with the left inferior frontal cortex—where Broca's area is located –, left supplementary motor area (SMA), left premotor cortex (BA6), and left insula. The phonological store has been shown to be associated with the left BA 40, corresponding to the left supramarginal gyrus located in the left inferior parietal lobule. Thus, these regions are essential for any kind of vWM task (Paulesu et al., 1993; Smith and Jonides, 1998; Henson et al., 2000; Buchsbaum and D'Esposito, 2008). Moreover, parietal activation has been interpreted as a buffer for modality-specific information. Whereas, the relevance of prefrontal and parietal regions for vWM has long been recognized, the cerebellum came into focus only some years ago. Originally regarded mainly as a structure involved in motor control and coordination, its involvement in higher-order cognitive processes, such as vWM, is no longer called into question (Ravizza et al., 2006; Hayter et al., 2007; Cooper et al., 2012; Thürling et al., 2012; Tomlinson et al., 2014). More specifically, it has been suggested that the cerebellum plays a relevant role in subvocal rehearsal, but the specific contribution of the cerebellum to the various processes involved in vWM is still a matter of debate (Desmond et al., 2003; Pleger and Timmann, 2018). Like the cerebellum, the basal ganglia (BG) are critical structures for motor control by enhancing desired motor behaviors and suppressing undesired ones (Alexander et al., 1986; Mink, 1996). In addition, the BG are involved in various cognitive processes, such as language production and working memory (McNab et al., 2008). Again, for many years, fMRI studies on vWM tended to focus on cortical structures such as parietal and frontal regions, underestimating the relevance of BG structures such as caudate, putamen and globus pallidus. Finally, limbic areas, such as cingulate, are known to be involved in vWM, but its contribution has likewise long been underestimated (Moore et al., 2013).

### Influencing Factors in the Neural Correlates of vWM

Activation in these brain regions can be influenced by several factors, such as age, gender, and type and difficulty (i.e., WM load) of the fMRI task. Moreover, the activation can be assumed to depend on individual performance (e.g., response velocity/speed as assessed by mean response times) and the statistical threshold which analyses are based on.

### Age

Older adults compared to younger adults have been found to show a more bilateral pattern of prefrontal cortex activity under comparable task demands, a finding which constituted the basis of the Hemispheric Asymmetry Reduction in Older Adults (HAROLD) model (Cabeza, 2002; Cabeza et al., 2004). One hypothesis is that bilateral activity in older adults could reflect a functional compensatory mechanism, in which age-related asymmetry reductions compensate neurocognitive decline leading to a less lateralized brain activity. This is known as the compensation view. A second hypothesis is the so-called dedifferentiation view which assumes a less specific recruitment of neural networks due to gradual changes occurring with age. In a PET study, Reuter-Lorenz et al. (2000) showed that PFC activity in younger adults was left lateralized for verbal and right lateralized for spatial stimuli, whereas older adults presented a bilateral PFC activation for verbal and visual tasks. This model is not only supported by functional neuroimaging results but also by behavioral results from a letter matching task (Reuter-lorenz et al., 1999). Apart from these models, a number of other theories related to age differences in brain activation have been proposed, such as the Posterior-Anterior Shift in Aging (PASA). This theory assumes both frontal over-activation and posterior midline cortex under-activation in older adults compared to younger ones (Davis et al., 2009). The Compensation-Related Utilization of Neural Circuits Hypothesis (CRUNCH) proposes that people will activate more cortical regions if task difficulty increases (Reuter-Lorenz and Cappell, 2008). Finally, the Scaffolding Theory of Aging and Cognition (STAC and STAC-r) suggests that the increased frontal activation with age is a marker of the adaptive human brain indicating a compensation for the structural and functional decline going along with aging (Park and Reuter-Lorenz, 2009). This theory takes a holistic view by considering compensation a normal process involved in our daily lives in order to be able to achieve our goals.

### Gender

The influence of gender in the context of WM and, more specifically, vWM, is still rather controversial, with some studies reporting no gender effects (Bell et al., 2006; Schmidt et al., 2009) and others reporting significant differences between male and female participants (Lejbak et al., 2011; Zilles et al., 2016). The controversial results might be due to the potential influence of sex hormones, which have been shown to influence several cognitive functions including vWM (Mordecai et al., 2008; Joseph et al., 2012). Sex hormones are known to fluctuate with, for instance, menstrual cycle or hormonal contraception. However, most studies did not provide any information on these aspects which may explain the result heterogeneity to some degree.

### Additional Factors (Tasks, Load, Mean, Reaction Time)

A previous meta-analysis showed differences in brain activity due to WM task type (Rottschy et al., 2012). They found that n-back and Sternberg tasks, which are typical fMRI WM tasks, not only showed differences in mental processes but also in brain activation. Moreover, tasks can vary in their difficulty through modulating the WM load (i.e., the number of items that need to be remembered). Load effects reflect the neural activation related to the increasing memory demands of information (Cowan et al., 2012; Cowan, 2017). Rottschy et al. (2012) found that load effects were mainly associated with activation in the bilateral inferior frontal gyrus. Finally, Honey et al. (2000) demonstrated that prolonged mean reaction times (RT) in response to a vWM task could influence activation in WM related brain regions. Therefore, these findings suggest that all the previously mentioned potential factors should be taken into consideration.

### Aim of the Study

Against this background, the first aim of the present study was to provide an updated and extended meta-analysis of the neural correlates of vWM in healthy humans using a coordinate-based meta-analysis. The second aim was to find out more about the role of the potential moderators (age, gender, type and difficulty of the fMRI task, mean RT, and statistical threshold). Although task performance which is related to the difficulty level could be another potential factor, it was not taken into consideration due to the heterogeneous assessment in the selected studies (i.e., absolute correct values, percentage of correct values, accuracy) as pointed also by Meule (2017). To our knowledge this is the first meta-analysis to study these factors in vWM. A better knowledge about their influence on the neural correlates of vWM will increase understanding of the general mechanisms of vWM as well as help to improve methods and analyses of future vWM studies.

## Materials and Methods

### Literature Search and Inclusion of Studies

**Figure 1:**
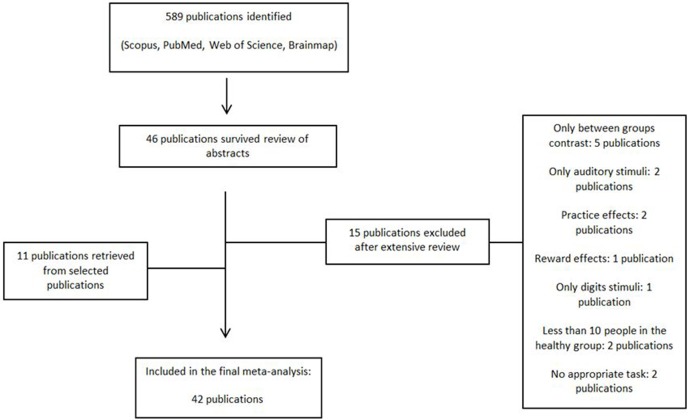
Flowchart diagram of selected papers.

An exhaustive literature search was conducted on whole-brain fMRI studies on vWM from January 2000 to December 2017. We searched the databases PubMed, Scopus and Web of Science for English-language studies with the combination of the following key words: “n-back,” “DMTS,” “Sternberg,” ”delayed matched to sample,” “delayed match to sample,” plus “verbal working memory,” “fMRI,” “healthy.” The Brainmap database was also searched with their respective search criteria (Subjects Size is more than 10, Experiments Paradigm Class is Delayed Match to Sample/n-back, Experiments Imaging Modality is fMRI, Conditions Stimulus is Visual Letters, and Subjects Handedness is Right). Further studies (11 publications) were identified through chasing citations from the selected studies (see Figure 1 for flowchart diagram). The “Meta-analysis of Observational Studies in Epidemiology” (MOOSE) guidelines Stroup et al. (2000) were used for the literature search and selection of studies. All articles were identified, selected and coded by a single investigator (M.E.). The same investigator double-checked the manually extracted peak coordinates and effect size values from the selected studies.

The criteria for inclusion were whole-brain analyses with reported results in a standard reference space (Talairach or MNI), inclusion of more than 10 healthy subjects and studies with clear boundaries between inclusion and exclusion criteria. Studies were excluded if they only included region-of-interests (ROI) analyses, did not report peak coordinates, investigated between- or within-group effects of pharmacological treatment, disease, living conditions, or used reward trials or emotional retrieval. We also excluded studies that re-analyzed previously reported data to avoid overestimating the effects. Positron emission tomography (PET) experiments were also not included in this meta-analysis due to methodological differences (e.g., differences in temporal resolution between PET and MRI) and differences in the underlying physiology (i.e., BOLD contrast vs. glucose mechanism).

### Comprehensive Meta-Analysis

We first conducted a meta-analysis of all the vWM studies. The demographic and study characteristics are shown in Table 1. The vast majority of the selected studies used the SPM software (https://www.fil.ion.ucl.ac.uk/spm/software/) to perform their fMRI analyses (83.3% of studies) indicating a clear bias toward this software package. Coordinates and *t*-values included in the analysis are shown in Supplementary Table 1. When only *p*- or *z*-values were reported, they were transformed into *t*-values taking into account the sample size per study. The influence of gender (% female), mean age, type of fMRI task (DMTS including the Sternberg task or n-back), mean RT, and the type of threshold used in the study (uncorrected vs. corrected) were studied with meta-regressions. The majority of studies corrected for multiple comparisons by controlling the false-discovery rate (FDR), except for one study that used the family-wise error rate (FWE) and another one that used Bonferroni correction. Those studies presenting results with peak coordinates at *p* < 0.005 or *p* < 0.001 uncorrected (33.3%) controlled for the cluster-size with different thresholds (5, 8, 10, 17, or 25 contiguous voxels). On a side note—although IQ and years of education have been shown to be associated with WM performance (Fukuda et al., 2010; Boller et al., 2017), we could not assess these factors here because the majority of studies did not provide any information on IQ or years of education.

**Table 1:** Characteristics of the 42 fMRI studies included in the meta-analysis.

	**Demographic data**				**fMRI task**				
**References**	***N***	**Mean age (age range)**	**SD age**	**% Fem**	**Task**	**Contrast**	**Phases**	**Mean RT (ms)**	**Mean accuracy (%)**
Altamura et al., 2007	18	27.4 (NA)	NA	38.9	Sternberg	Modulated by load and delay^†^, load alone^‡^	Block design	999.2	87.83
Bunge et al., 2001	16	27.0 (18–40)	NA	18.8	Sternberg	Load 6 > load 4	E,M,R	NA	93
Cabeza et al., 2002	20	22.6 (NA)	3.7	35	DMTS	WM > baseline	E.M,R	1486	91.6
Cairo et al., 2004	18	27.5 (NA)	NA	55.6	Sternberg	Average across loads^†^, Linear regression with load^‡^	E,M,R	NA	NA
Caseras et al., 2006	12	33.5 (24–45)	7.1	66.7	n-back	Modulated by load		635.8	89.83
Chen and Desmond, 2005a	17	28.6 (NA)	7.4	52.9	Sternberg	High load > low load (6 letters > 1 letter)	E,M,R	NA	84.6
Chen and Desmond, 2005b	15	22.5 (18–28)	2.7	46.7	Sternberg	High load > low load (6 letters > 2 letters)^†^	E,M,R	NA	88.5
Deckersbach et al., 2008	17	25.6 (NA)	5.9	100	n-back	2 > baseline		787.6	94.43
Desmond et al., 2003	13	55.6 (NA)	11.3	0	Sternberg	High load > low load (6 letters > 1 letter)	E,M,R	NA	NA
Dima et al., 2014	40	31.5 (NA)	10.4	50	n-back	1 > control, 2 > control, 3 > control^†^		1: 596 2: 659 3: 748	1 : 100 2: 91.2 3: 72.8
Garrett et al., 2011	19	34.9 (NA)	12.5	31.6	n-back	1 > control, 2 > control^†^		558.2	97.26
Gruber et al., 2010	18	33.9 (NA)	11.5	61.1	DMTS	Task > control	E,M,R	NA	91.9
Honey et al., 2000	20	39.3 (NA)	13.6	0	n-back	2 > control		560	96
Johnson et al., 2006	18	37.4 (NA)	11.5	16.7	Sternberg	Modulated by load^†^	E,R	995	92.45
Karlsgodt et al., 2005	13	24.1 (NA)	3.5	53.8	DMTS	WM > baseline	E,M,R	843.3	95.2
Kirschen et al., 2010	16	21.7 (NA)	6.0	31.3	Sternberg	High load > low load (6 letters > 2 letters)	E,M,R	NA	NA
Knops et al., 2006	16	27.0 (NA)	7.7	0	n-back	2 > 1		983.5	NA
Lim et al., 2008	12	68.6 (NA)	6.2	58.3	n-back	1 > baseline		650	96.9
Lythe et al., 2012	20	26.7 (NA)	6.7	0	n-back	Activation with increasing load		722	88.1
Marquand et al., 2008	20	43,7 (NA)	8.3	65	n-back	2 > control		NA	NA
Marvel and Desmond, 2010	16	23.7 (19–28)	NA	62.5	Sternberg	Task > baseline	E,M,R	NA	NA
McMillan et al., 2007	14	25.6 (NA)	3.6	64.3	n-back	2 > control: identification, 2 > control: color^†^		1562.5	78
McNab et al., 2008	11	24 (22–34)	4.0	63.6	Sternberg	Task > control	E,M,R	1460	91.3
Meisenzahl et al., 2006	12	33.6 (22–48)	9.27	8.3	n-back	2 > control		752	NA
Monks et al., 2004	12	45.6 (NA)	3.5	0	Sternberg	All levels	E,M,R	1080	90
Monks et al., 2004	12	45.6 (NA)	3.5	0	n-back	2 > control		NA	99.31
Mu et al., 2005	33	28.6 (18–45)	6.6	0	Sternberg	Task > control	E,M,R	621	NA
Narayanan et al., 2005	12	20.6 (19–26)	NA	41.7	Sternberg	WM > baseline	E,M,R	NA	NA
Norbury et al., 2014	15	38.3 (21–61)	NA	33.3	n-back	Tasks > control		932.6	NA
Ragland et al., 2002	11	32.2 (21–53)	NA	54.5	n-back	1 > control, 2 > control^†^, 2 > 1^‡^		NA	NA
Ravizza et al., 2004	10	24.8 (NA)	4.5	50	n-back	3 > control		NA	NA
Ravizza et al., 2004	11	NA (NA)	NA	NA	n-back	3 > control		NA	NA
Scheuerecker et al., 2008	23	32.6 (NA)	9.9	17.4	n-back	2 > control		751	NA
Schlösser et al., 2008	41	29.2 (NA)	8.9	34.1	Sternberg	Alphabetize > forward	E,M,R	1700.4	88.3
Schmidt et al., 2009	25	34.4 (18–58)	13.2	0	n-back	Task > control		670	83.84
Schmidt et al., 2009	21	33.1 (18–58)	12.3	100	n-back	Task > control		673.3	88.92
Seo et al., 2012	22	38.3 (NA)	8.5	100	n-back	2 > control		966.5	95.5
Valera et al., 2005	20	33.0 (18–55)	10.6	40	n-back	2 > control		843	90.2
Veltman et al., 2003	21	22.7 (NA)	3.6	66.7	Sternberg	Modulated by load	E,M,R	790	94.7
Veltman et al., 2003	21	22.7 (NA)	3.6	66.7	n-back	Modulated by load		715	97.7
Walter et al., 2003	13	27.1 (NA)	4.7	61.5	n-back	2 > control: identification, 2 > control: color^†^		NA	NA
Walter et al., 2007	17	30.9 (NA)	8.8	47.1	Sternberg	L1 > control, L2 > control, L3 > control^†^	E,M,R	L1: 760 L2: 873 L3: 1020	L1:93.2 L2: 90.9 L3: 87.1
Wishart et al., 2006	22	68.5 (25–75)	13.3	50	n-back	2 > control		NA	75.0
Wolf et al., 2006	15	28.1 (NA)	4.2	46.7	Sternberg	L2 > L1, L3 > L2^†^	E,M,R	L1:770.8 L2:882.0 L3:1034.5	L1: 95.5 L2: 92.6 L3: 93.0
Yan et al., 2011	28	20.9 (NA)	1.5	57.1	n-back	2 > control		617.4	95.9
Yoo et al., 2004	12	26.3 (20–36)	NA	33.3	n-back	2 > 1		NA	96.2

n, sample size; SD, standard deviation; NA, not announced; % Fem, percentage of female participants; L, level; E, encoding; M, maintenance; R, recall; RT, reaction time.

† Combination of several contrasts into the final study contrast.

‡ *Contrast selected for the load-effect meta-analysis*.

There are several established fMRI vWM paradigms: n-back, Sternberg, and delayed matching to sample (DMTS) tasks (Kirchner, 1958; Sternberg, 1966; Paule et al., 1998). N-back tasks include a sequential presentation of stimuli. Subjects have to decide whether the current stimulus is the same as the one *n* positions before (e.g., the previous one in a 1-back condition or the one two positions back in a 2-back condition). In Sternberg tasks, a set of stimuli is presented simultaneously that need to be maintained over a certain period which is followed by a single probe stimulus for which participants need to decide whether it was part of the set or not. In DMTS tasks, a single stimulus is presented. After the maintenance period, a set of multiple probes is presented from which participants need to recognize the single stimulus they had to memorize. While n-back tasks are normally presented in the form of a block-design, DMTS and Sternberg tasks are presented in an event-related design.

### Load-Effect Meta-Analysis

To assess the neural correlates of increasing vWM load (i.e., the difficulty of the fMRI task), we performed a load-effect meta-analysis. We only included studies in which there was a contrast between higher and lower vWM loads, such as 3-back vs. 1-back or 3-back vs. 2-back. The selected studies are shown in Supplementary Table 2.

### Meta-Analytical Approach: ES-SDM

We used the anisotropic effect-size version of seed-based d' mapping software (http://www.sdmproject.com) to conduct coordinate-based meta-analyses. The software uses a voxel-based meta-analytic approach. First, a strict selection of the reported peak coordinates of gray matter differences was applied by only including the studies containing whole-brain analyses. This is essential in order to avoid biased results from some neuroimaging studies, in which more liberal statistical thresholds were used for some ROIs relative to the rest of the brain. Peak coordinates in MNI or Talairach and effect size values were manually extracted from each contrast of interest in each study. All p- or z-values were transformed into *t*-values using SDM web utilities. Second, a map for the activation in gray matter was created for each study using the Automated Anatomical Labeling (AAL) atlas partitioned into 116 brain regions (Tzourio-Mazoyer et al., 2002). If a study included more than one contrast of interest, we adjusted for multiple contrasts by combining the created images of each contrast into one image for the final analyses. The ES-SDM software re-creates the maps from the studies by converting the *t*-value of each peak to Hedge's g (Alegria et al., 2016). Third, an anisotropic non-normalized Gaussian kernel was applied by assigning different values to the different neighboring voxels based on the spatial correlation between them (Radua et al., 2014). At the end, we obtained a mean map by a voxelwise calculation of the mean of the study maps, weighted by the square root of the sample size, so that studies with larger sample sizes contributed more strongly (Radua and Mataix-Cols, 2009).

To assess the robustness of the main findings, we performed a whole-brain Jackknife analysis. Jackknife analysis consists of repeating the statistical analyses several times by discarding one study each time thus demonstrating the stability of the results (Müller et al., 2018). Heterogeneity of effect sizes and publication bias were assessed with the *I*^2^ index and Egger's test (Egger et al., 1997; Müller et al., 2018). The *I*^2^ index provides the proportion of variability across studies that is due to true heterogeneity relative to that from sampling error (Higgins and Thompson, 2002). Egger's tests were used to test for asymmetry of funnel plots, serving as an indicator of publication bias (see Supplementary Figure 1 for examples).

Statistical significance was determined with random-effects models. We used the default threshold for the calculated mean (voxel-level *p* < 0.005 uncorrected, peak height threshold 1, minimum cluster extent 10 contiguous voxels) (Radua and Mataix-Cols, 2009). To control for multiple testing in the several meta-regressions we used a more conservative threshold, Bonferroni-corrected threshold of *p* < 0.001.

## Results

### Comprehensive Meta-Analysis (42 Studies)

**Figure 2:**
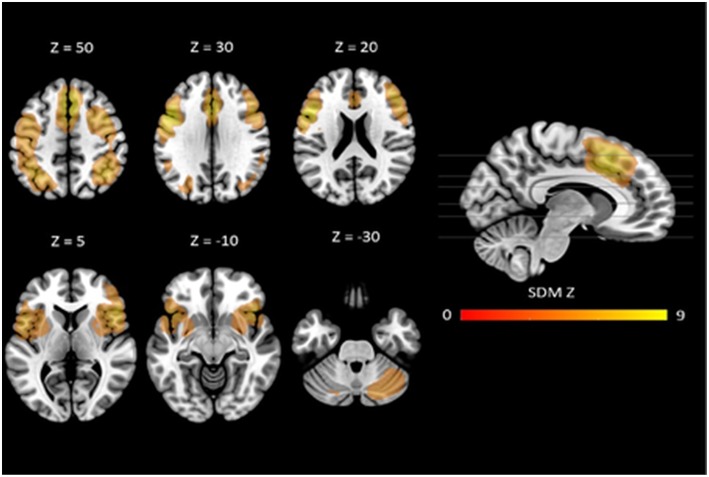
Neural correlates of vWM estimated by meta-analysis. Results are displayed at *p* < 0.005 (cluster size ≥10) projected on the MNI 151 T1 template.

The mean map of brain regions of the whole-brain meta-analysis for vWM is shown in Figure 2. The majority of studies reported only task-positive activation. We observed extended activation patterns in the frontal lobe including left superior frontal gyrus (SFG), medial frontal gyrus, right middle frontal gyrus (MFG), right inferior frontal gyrus (IFG), triangular, orbital and opercular part of the right IFG, orbital and opercular part of the left IFG, bilateral SMA, bilateral precentral gyrus, and left rolandic operculum. There was also activation in parietal areas including left post-central gyrus, right angular gyrus, and left inferior parietal gyri (IPG). Moreover, there was activation in the bilateral median cingulate, the left insula, the right lenticular nucleus (i.e., putamen and pallidum) and in bilateral cerebellum (crus I).

Robustness analyses showed that these results were preserved in all studies. Egger's tests indicated that there were some regions for which there was evidence of heterogeneity: left SFG, left SMA, left precentral gyrus, left post-central gyrus, right angular gyrus, left IPG, right median cingulate, left insula, and right cerebellum (crus I) (see Table 2).

**Table 2:** Comprehensive meta-analysis results.

**MNI coordinates**	**SDM-Z**	***p*-value**	**Region**	**Voxels**	**I^2^**	**JK**	**Egger test (*p*-value)**
−50,12,28	8.985	<0.00005	L. inferior frontal gyrus, opercular part	758	51.98	45/45	0.374
−46,8,36	8.831	<0.00005	L. precentral gyrus	1807	58.65	45/45	0.001
4,18,44	8.534	<0.00005	R. median cingulate / paracingulate gyri	631	59.46	45/45	0.015
4,24,46	8.483	<0.00005	R. supplementary motor area	784	55.79	45/45	0.051
0,18,40	8.359	<0.00005	L. superior frontal gyrus, medial	772	55.76	45/45	0.027
−2,8,36	8.322	<0.00005	L. median cingulate / paracingulate gyri	510	4.06	45/45	0.314
−2,22,46	8.214	<0.00005	L. supplementary motor area	1166	62.59	45/45	0.020
50,26,2	7.580	<0.00005	R. inferior frontal gyrus, triangular part	1246	0.00	45/45	0.732
50,18,8	7.397	<0.00005	R. inferior frontal gyrus, opercular part	888	3.29	45/45	0.168
40,−58,44	7.259	<0.00005	R. angular gyrus	873	23.80	45/45	0.001
46,24,−6	7.237	<0.00005	R. inferior frontal gyrus, orbital part	401	2.47	45/45	0.561
−36,−54,48	7.055	<0.00005	L. inferior parietal gyri	1804	45.38	45/45	0.000
40,6,50	6.917	<0.00005	R. precentral gyrus	1297	0.00	45/45	0.656
−44,0,16	6.293	<0.00005	L. rolandic operculum	428	10.86	45/45	0.386
26,6,50	5.911	<0.00005	R. middle frontal gyrus	1604	0.00	45/45	0.083
−42,18,−6	5.724	<0.00005	L. inferior frontal gyrus, orbital part	446	54.76	45/45	0.000
−48,−22,46	5.496	<0.00005	L. post-central gyrus	1582	45.32	45/45	0.002
−36,8,0	5.006	<0.00005	L. insula	939	8.50	45/45	0.019
22,−76,−30	4.683	0.000005	R. cerebellum, crus I	1186	45.55	45/45	0.009
32,0,−10	4.249	0.000107	R. lenticular nucleus, putamen	577	0.77	45/45	0.086
24,0,−6	4.167	0.000177	R. lenticular nucleus, pallidum	32			
−20,−78,−30	3.641	0.002827	L. cerebellum, crus I	36			

*Only one local peak per gray matter regions is displayed. Robustness analyses displayed for clusters>100 voxels (as in Fullana et al., 2018). MNI, Montreal Neurological Institute; SDM, signed differential mapping; I^2^, percentage of variance attributable to study heterogeneity; JK, jackknife sensitivity test; L., left; R., right*.

**Figure 3:**
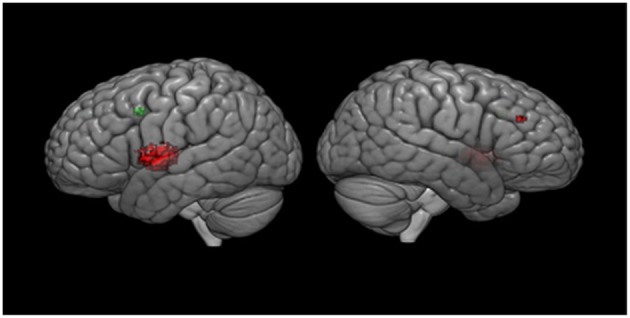
Meta-regression results. Results are displayed at *p* < 0.001 on MNI 152 2009. Red color, age regressor results; Green color, RT regressor results.

Meta-regression analyses confirmed that mean age and mean RT moderated activation in some brain regions. Mean age was associated with decreased activation in the left rolandic operculum, left insula, left superior temporal gyrus (STG), left IFG (opercular part), left heschl gyrus, left post-central gyrus, left lenticular nucleus (putamen), and the right MFG. Mean RT was positively associated with activation in the left precentral gyrus and the left MFG (see Figure 3 and Table 3). None of the other meta-regression analyses yielded any significant results.

**Table 3:** Meta-regression analysis.

**Mean_age**	**Clusters showing a negative correlation with age**	**Maximum**				**Cluster**	
	**MNI Coordinates**	**SDM value**	***p*-value**	**Voxels**	**Description**	**Breakdown**	**Voxels**
	−48,−4,8	−2.387	<0.00005	1204	L. rolandic operculum	L. rolandic operculum	415
						L. insula	332
						L. superior temporal gyrus	175
						L. inferior frontal gyrus, opercular part	115
						L. heschl gyrus	47
						L. post-central gyrus	34
						L. temporal pole, superior temporal gyrus	16
						L. lenticular nucleus, putamen	13
						L. precentral gyrus	1
						(undefined)	56
	48,38,24	−1.736	0.00028	16	R. inferior frontal gyrus, triangular part	R. middle frontal gyrus	10
						R. inferior frontal gyrus, triangular part	6
**Mean_RT**	**Clusters showing a positive correlation with RT**	**Maximum**				**Cluster**	
	MNI Coordinates	SDM value	p value	Voxels	Description	Breakdown	Voxels
	−46,10,42	3.949	0.00028	29	L. precentral gyrus	L. middle frontal gyrus	17
						L. precentral gyrus	12

*MNI, Montreal Neurological Institute; SDM, signed differential mapping, R., right; L., left*.

### Load-Effect Meta-Analysis (16 Studies)

We found activation in several frontal areas: right SFG (dorsolateral and medial part), left SFG (medial part), right MFG, right IFG (triangular part), left IFG (triangular and opercular part), right SMA, bilateral precentral gyrus, bilateral rolandic operculum. Moreover, there was activation in several parietal areas (left post-central gyrus, left angular gyrus, left SPG, and bilateral IPG) as well as in the left anterior cingulate gyri, bilateral median cingulate gyri, left fusiform gyrus, and right cerebellum (crus I and hemispheric lobule VI) (see Table 4).

**Table 4:** Load-effect meta-analysis results.

**MNI coordinates**	**SDM-Z**	***p*-value**	**Region**	**Voxels**	**I**^**2**^	**JK**	**Egger test (*p*-value)**
−46,8,38	5.617	<0.0000001	L. precentral gyrus	1315	52.15	15/15	0.152
46,34,18	5.502	<0.0000001	R. middle frontal gyrus	1378	31.70	15/15	0.339
8,32,48	5.442	<0.0000001	R. superior frontal gyrus, medial	387	5.78	15/15	0.828
−48,14,26	5.395	<0.0000001	L. inferior frontal gyrus, triangular part	1211	55.42	15/15	0.107
−50,16,22	5.356	<0.0000001	L. inferior frontal gyrus, opercular part	757	47.91	15/15	0.170
8,24,48	5.231	<0.0000001	R. supplementary motor area	452	29.95	15/15	0.741
0,28,50	5.201	<0.0000001	L. superior frontal gyrus, medial	892	38.10	15/15	0.864
−2,6,36	4.902	<0.0000001	L. median cingulate / paracingulate gyri	429	7.39	15/15	0.746
4,6,38	4.879	<0.0000001	R. median cingulate / paracingulate gyri	587	0.00	15/15	0.831
−40,−58,46	4.793	<0.0000001	L. angular gyrus	120	0.00	15/15	0.415
−2,8,30	4.686	<0.0000001	L. anterior cingulate / paracingulate gyri	633	4.28	15/15	0.687
50,30,4	4.646	<0.0000001	R. inferior frontal gyrus, triangular part	1228	0.59	15/15	0.534
−38,−48,44	4.563	0.00000001	L. inferior parietal gyri	961	39.39	15/15	0.487
22,−80,−30	4.248	0.00000101	R. cerebellum, crus I	1443	8.61	15/15	0.883
40,−46,48	4.143	0.00000179	R. inferior parietal gyri	682	62.41	15/15	0.001
22,14,56	3.676	0.00002283	R. superior frontal gyrus, dorsolateral	94			
−50,−16,42	3.640	0.00002819	L. post-central gyrus	688	1.97	15/15	0.790
52,10,−2	3.434	0.00009483	R. rolandic operculum	144	11.73	14/15	0.700
30,−50,−34	3.401	0.00011838	R. cerebellum, hemispheric lobule VI	704	7.75	15/15	0.974
−50,−6,14	3.354	0.00015760	L. rolandic operculum	347	0.20	15/15	0.827
−26,−60,54	3.242	0.00029481	L. superior parietal gyrus	255	0.00	15/15	0.343
46,−10,46	3.160	0.00046253	R. precentral gyrus	348	0.00	15/15	0.624
−32,−76,−16	2.933	0.00154328	L. fusiform gyrus	320	0.55	14/15	0.725

*Only one local peak per gray matter regions is displayed. Robustness analyses displayed for clusters>100 voxels (as in Fullana et al., 2018). MNI, Montreal Neurological Institute; SDM, signed differential mapping; I^2^, percentage of variance attributable to study heterogeneity; JK, jackknife sensitivity test; R., right; L., left*.

Jackknife analyses showed that the findings were preserved across studies, except for the right rolandic operculum and left fusiform gyrus, which were no longer detectable after discarding two papers. We only observed heterogeneity in the right inferior parietal gyrus (see Table 4).

## Discussion

The present comprehensive meta-analysis across 42 whole-brain vWM fMRI tasks showed vWM processing to be based on a fronto-parieto-cerebellar network and to involve also subcortical regions such as the cingulate, left insula and right lenticular nucleus. Thus, the present results corroborate previously discussed networks, but also provide evidence for the involvement of additional regions that have been neglected in the past in the discussion of vWM processing.

### Dual-Selection Model

A tentative explanation of the results is provided by the dual-selection model. Nee et al. (2013) proposed this model based on a meta-analysis of 36 event-related fMRI studies aimed at understanding the executive processes of WM. According to this model the caudal superior frontal sulcus (SFS) is associated with a spatial selection while the mid-lateral PFC is especially sensitive to non-spatial content, matching the “where” and “what” based selections, respectively. This proposal was further corroborated by a previous meta-analysis of 24 experiments based on an n-back task (Owen et al., 2005). The results of the present meta-analysis lend further support to the dual-selection model given that we also found activation of the mid-lateral PFC (bilateral IFG, right MFG, and medial part of the left SFG). The left SFG appeared to be a heterogeneous region. The fact that we found a noticeable bilateral prefrontal activation in this meta-analysis suggests that the assumption of a strongly left-lateralized verbal WM activation in PFC should be reconsidered. However, it needs to be clarified that we did not include studies systematically comparing spatial vs. non-spatial WM. Therefore, these conclusions need to be drawn with caution because the mere fact that we found the same activation does not fully support the dual-selection model.

### Phonological Loop

We did not find activation of the left supramarginal gyrus, which is known to be important for the phonological store, but instead in the region where the supramarginal gyrus is located, the left inferior parietal cortex. The activation of this region was found to be heterogeneous, which tends to be in line with the hypothesis of Buchsbaum and D'Esposito (2008). They argue that the phonological store does not precisely correspond to a single specific functional brain region, but rather is associated with several brain regions that underlie neural processes from perception and production of speech. Surprisingly, the present meta-analysis did not reveal any activation in the Wernicke area although this is an essential area for the comprehension and/or production of verbal material (Binder, 2015). This area is assumed to comprise mainly the posterior part of the superior temporal gyrus as well as the occipito-parieto-temporal junction including the angular gyrus. However, the exact location of the Wernicke area is still a matter of debate also due to its comprehensive and partly heterogeneous functionality in the context of verbal processing. Moreover, the fact that we used the AAL atlas, which comprises relatively large brain regions, might also explain why we were not able to isolate activation of this specific and somewhat ill-defined region. The fact that we did not find any activation in the right parietal cortex was also expected, since this region is assumed to serve spatial rehearsal. Hence, as opposed to the bilateral activation in the prefrontal cortex, activation in the parietal cortex turned out to be strongly left-lateralized, presumably due to modality. As predicted, we also found activation in the left IFG containing the Broca's area, as well as in the left SMA, which are components central to the articulatory loop. The fact that we did not find any activation in the premotor cortex could also be due to the atlas used. The AAL atlas does not contain this region because the labeled SMA embeds both the premotor cortex and the pre-SMA. Therefore, we cannot exclude that there was specific activation of the premotor cortex. In addition, there was activation in the left rolandic operculum, which is caudally adjacent to Broca's area. It has been demonstrated that this brain area is involved in speech production (Koelsch et al., 2009) and speech prosody processing (Wu et al., 2017). To the best of our knowledge, this is the first WM meta-analysis isolating activation specifically in this area. This finding may indicate that the majority of the participants used an overt rehearsal strategy during the vWM tasks. Further studies testing the impact of the opportunity for rehearsal during vWM tasks on brain activation are however needed to confirm this hypothesis.

### Attentional Control

Many conceptualizations of WM include an attentional control mechanism that supports dealing with interferences such as from other items in memory. The right IFG has been proposed to be an important region for attentional control (Aron et al., 2003; Forstmann et al., 2008). Specifically, Aron et al. 's (2003) data strongly suggest that response inhibition is uniquely located in the right IFG, in particular in its triangular part. The data were acquired by studying patients with lesions of the right frontal lobe during a go/no-go task. Forstmann et al. (2008) found a direct linkage between structural and functional properties of the right IFG, and its role in response inhibition. Another fMRI study (Aron and Poldrack, 2006) found that the IFG targets the subthalamic nucleus (STN) and regions in its vicinity. The STN sends excitatory projections to the globus pallidus externus, which, in turn, suppresses the thalamo-cortical output; this is assumed to lead to an inhibition of the initiated response. Finally, a strongly right-lateralized network comprising the right IFG, the STN, and also the pre-SMA, is recruited during response suppression (Aron, 2007). It remains unclear, however, whether the right IFG triggers the STN directly or via the pre-SMA (Aron et al., 2014). We found a strong activation of the right IFG, especially in the triangular part, the right SMA—which also includes the pre-SMA in this atlas –, and the right pallidum, giving support to the idea that these areas constitute a network subserving response inhibition in the context of vWM processing. Indeed, a substantial body of behavioral research has found that attentional control as employed in response inhibition tasks is related to WM capacity (Kane and Engle, 2003; Unsworth and Engle, 2007, but see Rey-Mermet et al., 2019). Notably, heterogeneity analyses confirmed the stability of these networks indicating that activation in these regions is not likely due to a possible publication bias. However, it is important to mention that the selected studies did not manipulate attention. The fact that we found activation in the same areas that mediate response inhibition in other experimental contexts does not completely mean that they do so in the context of vWM.

In addition to the IFG, the angular gyrus has been found to be activated in the context of response inhibition (Wager et al., 2005). The angular gyrus is located in the posterior part of the inferior parietal lobule and has been found to be activated in a variety of tasks (Seghier, 2013). Some anatomical studies (Makris et al., 2005, 2009; Uddin et al., 2010) define the angular gyrus as an important seed point, given its strong interaction with temporo-frontal subsystems as well as regions such as hippocampus, caudate, and precuneus. It is a key component of the default-mode network and shows activation in most tasks demanding information retrieval (Spaniol et al., 2009; Kim, 2010). The role of this region in memory retrieval is plausible given its strong connectivity with the hippocampus. To the best of our knowledge, this is the first time that the right angular gyrus appears in a vWM meta-analysis. Considering that this region has been reported to be important for inhibition and retrieval we conclude that the activation of the angular gyrus in the present meta-analysis may predominantly reflect the employment of attentional control during information retrieval. Although we cannot exclude the possibility of this region's activity being found due to presence of publication bias in the selected literature. Further studies allowing for a separate analysis of the retrieval process are however needed to further explore this assumption.

### Cerebellar and Subcortical Activations

It has been shown that cerebellum is connected not only to motor areas, but also to prefrontal cortical areas (Schmahmann, 1996); this suggests an involvement of the cerebellum in higher-order cognitive processes. A distinct cross-cerebro-cerebellar circuitry for vWM has been proposed with predominant involvement of right cerebellum, especially the lobule VI (Ng et al., 2016). In accordance with this proposal, earlier studies already pointed at the relevance of the right cerebellum in the context of vWM. Using inhibitory continuous theta burst stimulation (cTBS) Tomlinson et al. (2014) found that participants were less accurate during a verbal version of the Sternberg task if a trial was preceded by a stimulation of the right cerebellar hemisphere. Moreover, patients with right-sided cerebellar lesions have been found to be impaired in verbal memory, whereas patients with left cerebellar lesions turned out to be slower in a visuospatial task (Hokkanen et al., 2006). All these findings suggest a lateralized function of the cerebellum with its right hemisphere contributing mainly to verbal and its left hemisphere to visuospatial processing. Moreover, a meta-analysis (Stoodley and Schmahmann, 2009) analyzing cerebellum neuroimaging studies found that regions involved in vWM studies overlap with those involved in language tasks which is in agreement with domain-specific storage modules as in Baddeley's model. It corroborates the idea that vWM is more right-lateralized with a strong activation occurring mostly at the junction lobule VI/Crus I. Our results showing a significantly stronger activation in the right cerebellum (crus I) support this hypothesis. A case study of a right cerebellar hemispherectomy in an 18-years-old patient reported that the patient suffered from a disproportionate impairment of the rehearsal system, while the phonological store was preserved (Silveri et al., 1998). This could be due to anatomical connections between Broca's area, left SMA, right lobule VI and crus I of the cerebellum (Schmahmann, 1991). However, in the present meta-analysis, we did not differentiate between those processes and, thus, we cannot further investigate whether the right cerebellum is mainly involved in rehearsal. Still, our analysis provides clear evidence for the relevance of the right cerebellum, especially crus I, in the context of vWM processing. Further studies disentangling the different vWM processes are warranted to elucidate the specific function of the right cerebellum in vWM.

Apart from the cerebellum, a number of additional subcortical areas are assumed to be relevant for vWM. Thus, basal ganglia regions, especially the caudate and the lenticular nucleus, have been found to be activated during encoding and maintenance phases during vWM tasks (Lewis et al., 2004; Chang et al., 2007; Moore et al., 2013). Although in the present meta-analysis basal ganglia activation was restricted to the right lenticular nucleus, it remains unclear whether the activation is ascribable to these processes. Again, we were unable to distinguish between the different vWM processes given the available data. Caudate, putamen and capsular regions are known to receive afferents from the left pre-SMA region, which is involved in vWM (Inase et al., 1999). Crosson et al. (2003) found that basal ganglia activity was accompanied by activation of the left pre-SMA during a word production task. They hypothesized that the increase of right basal ganglia activation serves to suppress the non-dominant right frontal cortex, whereas the increase of the left basal ganglia activation serves to enhance the language processing of the left dominant hemisphere. Against the background of these findings, the basal ganglia can be assumed to interact closely with the frontal cortex and to serve as a selective gating mechanism for the prefrontal cortex (Frank et al., 2001). From this perspective, the findings showing the basal ganglia to be active only during encoding and maintenance phases seem plausible, because selective gating plays a major role for these processes. However, as we did not study the phases separately, we cannot rule out that these activations also reflect attentional processes in addition to pure vWM processes. Moreover, six publications compared activation conditions with a simple baseline (e.g., fixation of a cross hair). Hence, we cannot rule out that some parts of the subcortical activation were due to motor activity (i.e., button press in the activation conditions vs. no button press in the baseline condition). In the present meta-analysis we also found left pre-SMA activation, but a conclusion about their influence on basal ganglia is unwarranted without any connectivity data. In addition to the pre-SMA activation, our meta-analysis demonstrated significant activation in the anterior cingulate which has been found to be activated during vWM tasks before (Bedwell et al., 2005; Narayanan et al., 2005). It should be noted that in the AAL atlas the significant cluster was labeled as median cingulate, which is part of the anterior cingulate. It is striking, however, that a majority of vWM did not find an involvement of the anterior cingulate. Hence, future studies should make an attempt to clarify the specific contribution of the different parts of the cingulate to vWM.

### Age, Load, and Mean RT as Influencing Factors

Age-related changes in vWM are not fully understood because of a lack of longitudinal data. A recent longitudinal study found the activation of left prefrontal cortex (i.e., MFG and parts of the IFG) to be reduced during a vWM manipulation task in older people (Rieckmann et al., 2017). Somewhat in accordance with this finding, the present meta-analysis demonstrated a negative association between activation in the left and right IFG—including Broca's area—and age. In addition, we found a negative association between activation in the right MFG and age. It is known that cortical thickness, surface area, and volume of this region decrease with age (Lemaitre et al., 2012) which may, to some degree, explain this finding. Moreover, the right MFG plays a central role in reorienting attention from exogenous to endogenous attentional control (Japee et al., 2015). Our results are in agreement with the ontogenetic model of brain development according to which those brain regions that are the last to mature are the first to be affected by aging (Raz et al., 2005). Of note, all the other regions exhibiting a negative association between activation and age were localized in the left hemisphere. We found this negative association in the left insula, which—as stated above—plays a relevant role in the context of rehearsal, in the left putamen, which is involved in the active filtering of irrelevant material allowing us to focus on relevant material (Moore et al., 2013) in the left rolandic operculum, important for overt rehearsal, and in the left superior temporal gyrus, parts of which are critically involved in phonological storage. The fact that these regions important for different vWM processes showed a negative association with age might explain why older people tend to exhibit worse vWM performance, although it should be kept in mind that we did not take into account any longitudinal data or individual subject performance. Since these associations were detectable mainly in the left hemisphere and age-related changes were not restricted to the right PFC, our results seem to speak against the HAROLD model (Cabeza et al., 2002). Overall, the present results do not provide any evidence for a decrease of this lateralization with age, as claimed in other studies (Reuter-Lorenz et al., 2000; Cabeza et al., 2002, 2004). The fact that we found a decreased frontal activation with increasing age could either mean that the brain is not as adaptive as proposed by the earlier discussed STAC model or indicate that task demands were too high for elderly people leading to a “breakdown” of frontal activation instead of a compensatory increase. In order to draw any further conclusion it would be helpful to study the activation of these regions in elderly people taking also into account their individual task performance (e.g., accuracy). However, only three studies selected for the meta-analysis specifically included older populations; thus, the age-range was clearly undersampled in the current meta-analysis and the power to reliably assess the influence of age was too low. This might also explain why our results do not seem to be in line with the HAROLD model. Therefore, more empirical data comparing older and younger populations are necessary in order to find out more about specific age differences in activation during vWM. A better understanding of these age-related differences would pave the way for creating more sophisticated methods to preserve or enhance cognitive function in elderly populations.

Höller-Wallscheid et al. (2017) hypothesized the decreased lateralization across the PFC to be independent of age, but to depend on the subjective difficulty of WM tasks. In line with this hypothesis we found a bilateral activation across the PFC in the load effect meta-analysis. Our results are also in accordance with the load effect meta-analysis performed by Rottschy et al. (2012). The CRUNCH model states that the extent of cortical activation depends on the task load. Our results support this model, since we found a positive correlation between activation in several cortical regions (e.g., frontal areas) and task load. Apart from PFC areas we also found activation in the parietal cortex (IPG and left SPG) to be influenced by load, as reported in a previous study by Braver et al. (1997). Likewise, activation of the right lobule VI and crus I of the cerebellum turned out to depend on the difficulty of the vWM tasks. This is in accordance with a previous study which showed these parts of the cerebellum to respond to changes in vWM load (Kirschen et al., 2005). As stated before, there are anatomical connections between these parts of the cerebellum and frontal areas. Hence, the increased input from frontal regions involved in the articulatory system during a load manipulation could also reflect the increased activation of the right cerebellum. In addition, we found an association between load and activation in the fusiform gyrus. Tsapkini and Rapp (2010) pointed out that lesions of the left fusiform gyrus were significantly associated with reading and spelling deficits. In light of this finding, the positive correlation between load and activation in the left fusiform gyrus in the present study might indicate that a majority of people may have used overt rehearsal as a strategy to cope with increasing task difficulty.

It has long been recognized that RT is sensitive to manipulations of any kind of WM load (Just and Carpenter, 1992). Therefore, RT can also be viewed as a measure of load. A previous study found a significant positive correlation between RT and fMRI signal in nine subjects in the MFG and the left IFG (Braver et al., 1997). The present meta-analysis partially corroborates these findings showing both the left MFG and the left precentral gyrus to be positively associated with RT. However, our results seem to contradict a study by Honey et al. (2000), which reported that posterior parietal cortical activation was predicted by a prolonged RT in a vWM task. Importantly though, as states earlier, activation in the parietal cortex is influenced by load, which—in turn is related to RT. Moreover, the present meta-analysis revealed a positive association between left precentral gyrus activation and RT. This finding is plausible considering that the left precentral gyrus constitutes a major part of the primary motor area and its activation is contralateral to the side of the hand movement. Hence, increased activation in the primary motor cortex might facilitate faster responding. It should be emphasized that RT information was not available for all studies. Moreover, RT depends on many other factors such as number of responses alternatives, type of discrimination or delay time. Therefore, results of this factor should be treated with caution.

Although we performed a meta-analysis in which only fMRI studies were included, there has been a previous meta-analysis in which they selected both fMRI and PET studies to isolate the neural correlates of human working memory (Wager and Smith, 2003). Although they found some support for left frontal cortex dominance in vWM tasks, this was only for tasks with low executive demand. These results support our finding regarding the lateralization, i.e., the higher the difficulty on the task, the less lateralization of PFC activation is to be expected.

Finally, we expected the type of fMRI paradigm to be a significant moderator as demonstrated in a previous meta-analysis (Rottschy et al., 2012). This expectation was not met by the data. This could be due to a strong overlap in task activation, with potentially existing subtle quantity differences being too weak to be significant. We also found that gender did not affect activation associated with vWM tasks. However, we cannot exclude that gender differences would emerge when controlling for effects of sex hormones. Hence, future studies are required that carefully consider these potentially confounding factors.

### Limitations

In the present meta-analysis we discussed a number of relevant networks based on fMRI activity, such as the attentional system, but we did not take into account brain connectivity. However, the localization of brain areas is just the first step toward a more comprehensive understanding of the neural correlates of vWM. Analyses based on temporal dynamics, such as EEG or single-unit recordings, are essential to build a more integrative view. Another limitation regards our cerebellum findings. There is strong reason to assume that we did not find any inferior cerebellum activation because some of the scans included in the present meta-analysis did not cover the whole cerebellum due to methodological limitations (e.g., trade-off between brain coverage and repetition time).

## Conclusions

We used a coordinate-based meta-analysis to integrate the current literature on vWM in healthy humans. We found activation of the established fronto-parietal network and the right cerebellum, especially crus I, and lobule VI. Our results support the dual-selection model, according to which a mid-lateral PFC activation occurs due to verbal input. Moreover, our results illustrate that we should not underestimate the activation of subcortical regions that play an important role for response inhibition. Age, mean RT, and load moderate vWM task activation and, thus, should be taken into consideration in future research. Especially the influencing factor of age should be further analyzed since the sample included in the present meta-analysis consists of primarily young people. Mean reaction time, moreover, could be influenced by many other factors. Further, more fine-grained studies are needed to gain a better understanding of the neural correlates underlying processes involved in vWM including encoding, maintenance, and retrieval.

## Author Contributions

ME, CB, and KK contributed to the conception and design of the study. ME selected the analyzed studies, performed the statistical analysis, and wrote the first draft of the manuscript. CB and KK wrote sections of the manuscript. All authors contributed to manuscript revision, read and approved the submitted version.

### Conflict of Interest Statement

The authors declare that the research was conducted in the absence of any commercial or financial relationships that could be construed as a potential conflict of interest.
