# Supplementary material for: Neural Correlates of Verbal Working Memory: An fMRI Meta-Analysis
Source: Front Hum Neurosci. 2019 Jun 12;13:180. doi: 10.3389/fnhum.2019.00180 (PMC6581736; doi:10.3389/fnhum.2019.00180)
Supplement: Supplementary file 1 [file Data_Sheet_1.docx]

Supplementary Material

# Supplementary Data

# Supplementary Figures and Tables

**Supplementary Table 1**. Coordinates used for the meta-analysis

Abbreviations: N: sample size; MNI: Montreal Neurological Institute; p: positive peak

|  | | | | | | | |  | |  |  |  |  | |  |  |  |  |
| --- | --- | --- | --- | --- | --- | --- | --- | --- | --- | --- | --- | --- | --- | --- | --- | --- | --- | --- |
|  | Study | Coordinate system | N | x | y | z | peak (t) | | Contrasts | | | | | Specificities | | | | |
| 1 | Altamura *et al.*, 2007 | MNI | 18 | -44 | 22 | 26 | 6.34 | | Main effect of load | | | | |  | | | | |
|  |  |  |  | 37 | 37 | 14 | 7.37 | |  | | | | |  | | | | |
|  |  |  |  | -51 | 5 | 27 | 12.29 | |  | | | | |  | | | | |
|  |  |  |  | 33 | 18 | -1 | 18.16 | |  | | | | |  | | | | |
|  |  |  |  | -44 | -45 | 35 | 10.35 | |  | | | | |  | | | | |
|  |  |  |  | 33 | -48 | 46 | 7.92 | |  | | | | |  | | | | |
|  |  |  |  | 1 | 20 | 48 | 9.57 | |  | | | | |  | | | | |
|  |  |  |  | -10 | 10 | 49 | 8.24 | |  | | | | |  | | | | |
|  |  |  |  | 33 | -1 | 55 | 11.44 | |  | | | | |  | | | | |
|  |  |  |  | -37 | -5 | 50 | 7.67 | |  | | | | |  | | | | |
|  |  |  |  | -11 | 8 | 54 | 8.11 | | Main effect of delay | | | | |  | | | | |
|  |  |  |  | -52 | 4 | 21 | 4.34 | |  | | | | |  | | | | |
|  |  |  |  | -51 | 4 | 21 | 4.34 | |  | | | | |  | | | | |
| 2 | Bunge *et al.*, 2001 | Talairach | 16 | -42 | -2 | 32 | 4.52 | | Load 6 > load 4 | | | | |  | | | | |
|  |  |  |  | -34 | 20 | 4 | 5.78 | |  | | | | |  | | | | |
|  |  |  |  | 36 | 24 | 4 | 5.96 | |  | | | | |  | | | | |
|  |  |  |  | 36 | 30 | -4 | 4.96 | |  | | | | |  | | | | |
|  |  |  |  | 38 | 46 | 0 | 3.68 | |  | | | | |  | | | | |
|  |  |  |  | 40 | 34 | 20 | 4.15 | |  | | | | |  | | | | |
|  |  |  |  | 48 | 26 | 28 | 4.12 | |  | | | | |  | | | | |
|  |  |  |  | 26 | 2 | 56 | 3.65 | |  | | | | |  | | | | |
|  |  |  |  | 8 | 20 | 44 | 3.97 | |  | | | | |  | | | | |
|  |  |  |  | 2 | 4 | 28 | 3.84 | |  | | | | |  | | | | |
|  |  |  |  | -28 | -70 | 36 | 4.59 | |  | | | | |  | | | | |
|  |  |  |  | 44 | -50 | 48 | 4.49 | |  | | | | |  | | | | |
|  |  |  |  | 16 | -46 | -16 | 3.87 | |  | | | | |  | | | | |
|  |  |  |  | 24 | -62 | -24 | 5.62 | |  | | | | |  | | | | |
| 3 | Cabeza *et al.*, 2002 | Talairach | 20 | -42 | 15 | 27 | 2.40 | | WM > baseline | | | | |  | | | | |
|  |  |  |  | -33 | 26 | 1 | 2.90 | |  | | | | |  | | | | |
|  |  |  |  | 31 | 26 | 1 | 2.40 | |  | | | | |  | | | | |
|  |  |  |  | 1 | 1 | 60 | 5.80 | |  | | | | |  | | | | |
|  |  |  |  | 1 | 1 | 56 | 4.70 | |  | | | | |  | | | | |
|  |  |  |  | -19 | -69 | 43 | 3.60 | |  | | | | |  | | | | |
|  |  |  |  | 24 | -72 | 43 | 5.90 | |  | | | | |  | | | | |
|  |  |  |  | -26 | -69 | 39 | 6.9 | |  | | | | |  | | | | |
|  |  |  |  | 1 | -43 | 44 | 4.3 | |  | | | | |  | | | | |
|  |  |  |  | -16 | -84 | -25 | 7.0 | |  | | | | |  | | | | |
|  |  |  |  | 21 | -84 | -25 | 6.9 | |  | | | | |  | | | | |
|  |  |  |  | -26 | -82 | -25 | 7.8 | |  | | | | |  | | | | |
|  |  |  |  | 21 | -80 | -25 | 6.8 | |  | | | | |  | | | | |
|  |  |  |  | -19 | -26 | -9 | 3.5 | |  | | | | |  | | | | |
|  |  |  |  | 21 | -26 | -9 | 2.0 | |  | | | | |  | | | | |
|  |  |  |  | -16 | -32 | -5 | 4.4 | |  | | | | |  | | | | |
|  |  |  |  | 18 | -32 | -5 | 2.5 | |  | | | | |  | | | | |
|  |  |  |  | 1 | 19 | 34 | 2.2 | |  | | | | |  | | | | |
|  |  |  |  | -9 | -22 | 6 | 10.4 | |  | | | | |  | | | | |
|  |  |  |  | 10 | -18 | 6 | 5.6 | |  | | | | |  | | | | |
|  |  |  |  | -16 | 4 | 3 | 3.8 | |  | | | | |  | | | | |
|  |  |  |  | 18 | 4 | 3 | 2.6 | |  | | | | |  | | | | |
|  |  |  |  | -3 | -84 | 2 | 12.9 | |  | | | | |  | | | | |
|  |  |  |  | 10 | -80 | 3 | 9.5 | |  | | | | |  | | | | |
|  |  |  |  | -6 | -87 | 2 | 6.4 | |  | | | | |  | | | | |
|  |  |  |  | 10 | -72 | 3 | 6.2 | |  | | | | |  | | | | |
|  |  |  |  | -42 | -11 | 46 | 7.8 | |  | | | | |  | | | | |
|  |  |  |  | -29 | -22 | 51 | 9.5 | |  | | | | |  | | | | |
|  |  |  |  | -42 | 8 | 27 | 4.5 | |  | | | | |  | | | | |
|  |  |  |  | -45 | 0 | 36 | 7.8 | |  | | | | |  | | | | |
|  |  |  |  | 24 | -7 | 52 | 3.7 | |  | | | | |  | | | | |
|  |  |  |  | -49 | -40 | 24 | 5.0 | |  | | | | |  | | | | |
|  |  |  |  | 7 | -65 | 39 | 2.6 | |  | | | | |  | | | | |
| 4 | Cairo *et al.*,2004 | Talairach | 18 | -12 | 2 | 56 | 17.68 | | Encoding | | | | | Average across all loads | | | | |
|  |  |  |  | -48 | -10 | 38 | 12.51 | |  | | | | |  | | | | |
|  |  |  |  | 36 | -16 | 56 | 9.36 | |  | | | | |  | | | | |
|  |  |  |  | -52 | 4 | 18 | 9.00 | |  | | | | |  | | | | |
|  |  |  |  | 52 | 6 | 26 | 8.46 | |  | | | | |  | | | | |
|  |  |  |  | 32 | 44 | 28 | 6.84 | |  | | | | |  | | | | |
|  |  |  |  | -44 | -40 | 42 | 8.46 | |  | | | | |  | | | | |
|  |  |  |  | 32 | -48 | 42 | 10.94 | |  | | | | |  | | | | |
|  |  |  |  | -24 | -68 | 36 | 12.51 | |  | | | | |  | | | | |
|  |  |  |  | 28 | -68 | 40 | 12.99 | |  | | | | |  | | | | |
|  |  |  |  | -48 | -62 | -14 | 9.66 | |  | | | | |  | | | | |
|  |  |  |  | 40 | -62 | 4 | 10.38 | |  | | | | |  | | | | |
|  |  |  |  | -32 | -92 | 8 | 9.25 | |  | | | | |  | | | | |
|  |  |  |  | 24 | -92 | 8 | 12.62 | |  | | | | |  | | | | |
|  |  |  |  | -24 | 4 | 0 | 13.10 | |  | | | | |  | | | | |
|  |  |  |  | 20 | 12 | 0 | 9.25 | |  | | | | |  | | | | |
|  |  |  |  | -4 | -30 | -6 | 9.66 | |  | | | | |  | | | | |
|  |  |  |  | -12 | -78 | -16 | 9.00 | |  | | | | |  | | | | |
|  |  |  |  | 28 | -64 | -18 | 9.51 | | Maintenance | | | | |  | | | | |
|  |  |  |  | -4 | 10 | 48 | 8.83 | |  | | | | |  | | | | |
|  |  |  |  | -44 | -6 | 52 | 6.56 | |  | | | | |  | | | | |
|  |  |  |  | -16 | 10 | 58 | 5.89 | |  | | | | |  | | | | |
|  |  |  |  | 20 | 10 | 54 | 6.07 | |  | | | | |  | | | | |
|  |  |  |  | -48 | 32 | 20 | 5.73 | |  | | | | |  | | | | |
|  |  |  |  | 36 | 48 | 24 | 6.31 | |  | | | | |  | | | | |
|  |  |  |  | -28 | -60 | 40 | 5.10 | |  | | | | |  | | | | |
|  |  |  |  | -20 | -94 | -2 | 6.43 | |  | | | | |  | | | | |
|  |  |  |  | 16 | -90 | 4 | 6.43 | |  | | | | |  | | | | |
|  |  |  |  | -24 | 12 | -8 | 5.18 | |  | | | | |  | | | | |
|  |  |  |  | 32 | 24 | -2 | 5.10 | |  | | | | |  | | | | |
|  |  |  |  | 28 | -64 | -24 | 6.38 | | Retrieval | | | | | Average across all loads | | | | |
|  |  |  |  | 4 | 14 | 44 | 9.86 | |  | | | | |  | | | | |
|  |  |  |  | -40 | -16 | 56 | 8.17 | |  | | | | |  | | | | |
|  |  |  |  | -48 | -4 | 8 | 7.47 | |  | | | | |  | | | | |
|  |  |  |  | -36 | 20 | -8 | 8.17 | |  | | | | |  | | | | |
|  |  |  |  | 32 | 26 | -4 | 8.17 | |  | | | | |  | | | | |
|  |  |  |  | -48 | -34 | 38 | 12.11 | |  | | | | |  | | | | |
|  |  |  |  | 32 | -44 | 46 | 7.71 | |  | | | | |  | | | | |
|  |  |  |  | -40 | -36 | 60 | 7.86 | |  | | | | |  | | | | |
|  |  |  |  | 56 | -18 | 34 | 7.88 | |  | | | | |  | | | | |
|  |  |  |  | 12 | -68 | 36 | 7.77 | |  | | | | |  | | | | |
|  |  |  |  | -56 | -66 | 4 | 9.07 | |  | | | | |  | | | | |
|  |  |  |  | 48 | -58 | 0 | 10.10 | |  | | | | |  | | | | |
|  |  |  |  | -16 | -12 | -6 | 10.10 | |  | | | | |  | | | | |
|  |  |  |  | 20 | -56 | -20 | 10.90 | |  | | | | |  | | | | |
|  |  |  |  | 0 | -58 | -8 | 10.63 | |  | | | | |  | | | | |
|  |  |  |  | 24 | -64 | -40 | 8.90 | |  | | | | |  | | | | |
|  |  |  |  | 12 | -70 | -20 | 8.69 | |  | | | | |  | | | | |
|  |  |  |  | 8 | -80 | -30 | 7.95 | |  | | | | |  | | | | |
| 5 | Caseras *et al.,* 2006 | Talairach | 12 | -4 | -70 | 40 | p | | Across n-back load levels | | | | | Control group | | | | |
|  |  |  |  | 40 | 37 | 23 | p | |  | | | | |  | | | | |
|  |  |  |  | 7 | -81 | -16 | p | |  | | | | |  | | | | |
|  |  |  |  | 43 | 22 | 36 | p | |  | | | | |  | | | | |
|  |  |  |  | 0 | -74 | 33 | p | |  | | | | |  | | | | |
|  |  |  |  | 29 | 7 | 59 | p | |  | | | | |  | | | | |
|  |  |  |  | 32 | 56 | 20 | p | |  | | | | |  | | | | |
|  |  |  |  | 4 | 22 | 40 | p | |  | | | | |  | | | | |
|  |  |  |  | 40 | -52 | 33 | p | |  | | | | |  | | | | |
|  |  |  |  | 51 | 26 | 20 | p | |  | | | | |  | | | | |
| 6 | Chen and Desmond, 2005a | Talairach | 17 | -39.6 | 19.1 | -6 | 6.21 | | High load > low load | | | | |  | | | | |
|  |  |  |  | -31.7 | -48.4 | 43 | 6.67 | |  | | | | |  | | | | |
|  |  |  |  | -39.6 | -37.2 | 33.2 | 5.56 | |  | | | | |  | | | | |
|  |  |  |  | -5.9 | 21 | 32.1 | 5.34 | |  | | | | |  | | | | |
|  |  |  |  | 41.6 | 18.6 | -16.1 | 7.52 | |  | | | | |  | | | | |
|  |  |  |  | 37.6 | 22.8 | -11.2 | 7.02 | |  | | | | |  | | | | |
|  |  |  |  | 33.7 | -40.7 | 40.7 | 5.78 | |  | | | | |  | | | | |
|  |  |  |  | 35.6 | 31.7 | 11.3 | 5.81 | |  | | | | |  | | | | |
|  |  |  |  | 29.7 | 44.8 | 3.3 | 4.75 | |  | | | | |  | | | | |
|  |  |  |  | 41.6 | 10.7 | 19.7 | 5.78 | |  | | | | |  | | | | |
|  |  |  |  | 23.8 | -72.2 | -45.2 | 4.79 | |  | | | | |  | | | | |
| 7 | Chen and Desmond, 2005b | Talairach | 15 | -46 | -76 | -11 | 10.28 | | Encoding | | | | |  | | | | |
|  |  |  |  | -48 | -59 | -12 | 10.28 | |  | | | | |  | | | | |
|  |  |  |  | -53 | -71 | -12 | 8.61 | |  | | | | |  | | | | |
|  |  |  |  | -46 | -80 | -4 | 8.25 | |  | | | | |  | | | | |
|  |  |  |  | -63 | 8 | 11 | 10.28 | |  | | | | |  | | | | |
|  |  |  |  | -51 | 0 | 44 | 10.28 | |  | | | | |  | | | | |
|  |  |  |  | -57 | -11 | 47 | 8.61 | |  | | | | |  | | | | |
|  |  |  |  | -51 | -2 | 37 | 7.90 | |  | | | | |  | | | | |
|  |  |  |  | -20 | -35 | -2 | 10.28 | |  | | | | |  | | | | |
|  |  |  |  | -26 | -37 | -7 | 9.40 | |  | | | | |  | | | | |
|  |  |  |  | -22 | -36 | -13 | 9.00 | |  | | | | |  | | | | |
|  |  |  |  | -32 | -30 | -10 | 7.57 | |  | | | | |  | | | | |
|  |  |  |  | -28 | -31 | -2 | 6.95 | |  | | | | |  | | | | |
|  |  |  |  | -6 | 3 | 51 | 6.66 | |  | | | | |  | | | | |
|  |  |  |  | -2 | 8 | 46 | 6.38 | |  | | | | |  | | | | |
|  |  |  |  | -10 | 3 | 68 | 6.38 | |  | | | | |  | | | | |
|  |  |  |  | -2 | 3 | 59 | 6.12 | |  | | | | |  | | | | |
|  |  |  |  | 24 | -33 | -2 | 10.28 | |  | | | | |  | | | | |
|  |  |  |  | 26 | -33 | -8 | 9.40 | |  | | | | |  | | | | |
|  |  |  |  | 18 | -10 | -3 | 7.57 | |  | | | | |  | | | | |
|  |  |  |  | 42 | -7 | 56 | 10.28 | |  | | | | |  | | | | |
|  |  |  |  | 55 | -7 | 50 | 10.28 | |  | | | | |  | | | | |
|  |  |  |  | 57 | -2 | 42 | 8.61 | |  | | | | |  | | | | |
|  |  |  |  | 34 | -5 | 63 | 8.25 | |  | | | | |  | | | | |
|  |  |  |  | 28 | -99 | 7 | 4.54 | |  | | | | |  | | | | |
|  |  |  |  | 24 | -98 | 14 | 5.54 | |  | | | | |  | | | | |
|  |  |  |  | 63 | 4 | 5 | 5.62 | |  | | | | |  | | | | |
|  |  |  |  | -18 | -27 | -32 | 6.67 | |  | | | | |  | | | | |
|  |  |  |  | -10 | -23 | -32 | 6.67 | |  | | | | |  | | | | |
|  |  |  |  | -24 | -33 | -34 | 6.12 | |  | | | | |  | | | | |
|  |  |  |  | 34 | -64 | -34 | 9.00 | |  | | | | |  | | | | |
|  |  |  |  | 46 | -86 | -16 | 6.66 | |  | | | | |  | | | | |
|  |  |  |  | 38 | -56 | -36 | 6.38 | |  | | | | |  | | | | |
|  |  |  |  | 38 | -67 | -29 | 6.12 | |  | | | | |  | | | | |
|  |  |  |  | 28 | -41 | -33 | 6.66 | |  | | | | |  | | | | |
|  |  |  |  | 0 | -79 | -21 | 5.62 | |  | | | | |  | | | | |
|  |  |  |  | 26 | -72 | -49 | 5.86 | | Maintenance | | | | |  | | | | |
|  |  |  |  | -12 | 4 | 48 | 10.28 | |  | | | | |  | | | | |
|  |  |  |  | -4 | 6 | 48 | 10.28 | |  | | | | |  | | | | |
|  |  |  |  | -61 | 9 | 20 | 7.90 | |  | | | | |  | | | | |
|  |  |  |  | -53 | 6 | 7 | 7.90 | |  | | | | |  | | | | |
|  |  |  |  | -55 | 7 | 25 | 7.57 | |  | | | | |  | | | | |
|  |  |  |  | -36 | 27 | -10 | 8.61 | |  | | | | |  | | | | |
|  |  |  |  | -40 | -47 | 41 | 9.00 | |  | | | | |  | | | | |
|  |  |  |  | -18 | 9 | -6 | 5.62 | |  | | | | |  | | | | |
|  |  |  |  | -10 | 11 | -7 | 5.39 | |  | | | | |  | | | | |
|  |  |  |  | -48 | -61 | -9 | 6.95 | |  | | | | |  | | | | |
|  |  |  |  | 28 | 25 | -6 | 5.38 | |  | | | | |  | | | | |
|  |  |  |  | 12 | 13 | -6 | 4.54 | |  | | | | |  | | | | |
|  |  |  |  | -16 | -24 | -22 | 4.94 | |  | | | | |  | | | | |
|  |  |  |  | 18 | -70 | -44 | 6.38 | | Recall | | | | |  | | | | |
|  |  |  |  | -4 | 31 | 28 | 7.90 | |  | | | | |  | | | | |
|  |  |  |  | -10 | 18 | 43 | 6.38 | |  | | | | |  | | | | |
|  |  |  |  | -6 | 0 | 48 | 6.38 | |  | | | | |  | | | | |
|  |  |  |  | -53 | 9 | 25 | 7.90 | |  | | | | |  | | | | |
|  |  |  |  | -61 | 9 | 24 | 7.25 | |  | | | | |  | | | | |
|  |  |  |  | -16 | -15 | 6 | 6.66 | |  | | | | |  | | | | |
|  |  |  |  | -36 | -5 | 61 | 7.57 | |  | | | | |  | | | | |
|  |  |  |  | -44 | 13 | -6 | 4.74 | |  | | | | |  | | | | |
|  |  |  |  | 50 | 13 | -11 | 6.12 | |  | | | | |  | | | | |
|  |  |  |  | 44 | 10 | -2 | 5.86 | |  | | | | |  | | | | |
|  |  |  |  | 6 | -19 | 18 | 6.95 | |  | | | | |  | | | | |
|  |  |  |  | -2 | -81 | -23 | 5.45 | |  | | | | |  | | | | |
| 8 | Deckersbach *et al.*, 2008 | MNI | 17 | -8 | 4 | -56 | 6.52 | | N-back > baseline | | | | | Control group | | | | |
|  |  |  |  | -58 | 6 | 22 | 4.40 | |  | | | | |  | | | | |
|  |  |  |  | 36 | 18 | -6 | 4.66 | |  | | | | |  | | | | |
|  |  |  |  | 26 | -8 | 46 | 4.49 | |  | | | | |  | | | | |
|  |  |  |  | 12 | -6 | 12 | 4.12 | |  | | | | |  | | | | |
|  |  |  |  | -46 | -4 | 42 | 6.26 | |  | | | | |  | | | | |
|  |  |  |  | -56 | -16 | 28 | 4.89 | |  | | | | |  | | | | |
|  |  |  |  | 24 | -10 | -2 | 5.45 | |  | | | | |  | | | | |
|  |  |  |  | -8 | -20 | -12 | 4.66 | |  | | | | |  | | | | |
|  |  |  |  | 68 | -12 | -6 | 5.13 | |  | | | | |  | | | | |
|  |  |  |  | -58 | -10 | -8 | 4.49 | |  | | | | |  | | | | |
|  |  |  |  | -32 | 18 | 2 | 6.24 | |  | | | | |  | | | | |
| 9 | Desmond *et al.,* 2003 | Talairach | 13 | -55 | 19 | -4 | 3.33 | | High load > low load | | | | | Control group | | | | |
|  |  |  |  | -36 | -69 | -17 | 3.75 | |  | | | | |  | | | | |
|  |  |  |  | -42 | 4 | 35 | 3.2 | |  | | | | |  | | | | |
|  |  |  |  | -36 | 24 | 21 | 3.97 | |  | | | | |  | | | | |
|  |  |  |  | -34 | 10 | -31 | 4.08 | |  | | | | |  | | | | |
|  |  |  |  | -28 | -49 | -46 | 3.37 | |  | | | | |  | | | | |
|  |  |  |  | -30 | -19 | -24 | 3.64 | |  | | | | |  | | | | |
|  |  |  |  | -4 | 23 | 34 | 3.24 | |  | | | | |  | | | | |
|  |  |  |  | 42 | -63 | -20 | 5.46 | |  | | | | |  | | | | |
|  |  |  |  | 40 | -72 | -18 | 5.27 | |  | | | | |  | | | | |
|  |  |  |  | 46 | -58 | -32 | 5.08 | |  | | | | |  | | | | |
|  |  |  |  | 0 | 7 | 64 | 6.18 | |  | | | | |  | | | | |
|  |  |  |  | 28 | -21 | -26 | 3.71 | |  | | | | |  | | | | |
|  |  |  |  | 36 | -45 | -47 | 4.46 | |  | | | | |  | | | | |
|  |  |  |  | 36 | -62 | -41 | 3.06 | |  | | | | |  | | | | |
|  |  |  |  | 48 | -6 | 32 | 3.03 | |  | | | | |  | | | | |
| 10 | Dima *et al.,* 2014 | MNI | 40 | 46 | -46 | 44 | 9.06 | | 1 > 0 back | | | | |  | | | | |
|  |  |  |  | -44 | -42 | 38 | 8.40 | |  | | | | |  | | | | |
|  |  |  |  | -46 | 32 | 30 | 8.64 | |  | | | | |  | | | | |
|  |  |  |  | 52 | 36 | 30 | 8.34 | |  | | | | |  | | | | |
|  |  |  |  | 32 | 6 | 62 | 7.70 | |  | | | | |  | | | | |
|  |  |  |  | 40 | -48 | 44 | 12.54 | | 2 > 0 back | | | | |  | | | | |
|  |  |  |  | -36 | -52 | 46 | 12.09 | |  | | | | |  | | | | |
|  |  |  |  | 46 | 32 | 28 | 10.08 | |  | | | | |  | | | | |
|  |  |  |  | -42 | 8 | 28 | 9.13 | |  | | | | |  | | | | |
|  |  |  |  | 8 | 18 | 48 | 9.70 | |  | | | | |  | | | | |
|  |  |  |  | -4 | 10 | 58 | 9.70 | |  | | | | |  | | | | |
|  |  |  |  | 34 | 24 | -2 | 8.75 | |  | | | | |  | | | | |
|  |  |  |  | 30 | 8 | 58 | 8.00 | |  | | | | |  | | | | |
|  |  |  |  | 50 | -42 | 42 | 12.93 | | 3 > 0 back | | | | |  | | | | |
|  |  |  |  | -48 | -48 | 48 | 12.80 | |  | | | | |  | | | | |
|  |  |  |  | -48 | 26 | 30 | 12.86 | |  | | | | |  | | | | |
|  |  |  |  | 48 | 40 | 30 | 12.86 | |  | | | | |  | | | | |
|  |  |  |  | -10 | 26 | 30 | 11.43 | |  | | | | |  | | | | |
|  |  |  |  | 8 | 20 | 28 | 11.43 | |  | | | | |  | | | | |
|  |  |  |  | -34 | 22 | 0 | 9.97 | |  | | | | |  | | | | |
|  |  |  |  | -12 | -10 | 6 | 8.68 | |  | | | | |  | | | | |
|  |  |  |  | -44 | 46 | 2 | 7.72 | |  | | | | |  | | | | |
|  |  |  |  | 34 | 20 | 0 | 7.13 | |  | | | | |  | | | | |
| 11 | Garrett *et al.*, 2011 | Talairach | 19 | 57 | 32 | 13 | 6.25 | | 1 > 0 back | | | | | Control group | | | | |
|  |  |  |  | 55 | 32 | 15 | 5.78 | |  | | | | |  | | | | |
|  |  |  |  | 36 | 8 | 49 | 5.65 | |  | | | | |  | | | | |
|  |  |  |  | -42 | -45 | 41 | 6.18 | |  | | | | |  | | | | |
|  |  |  |  | -42 | -43 | 37 | 5.56 | |  | | | | |  | | | | |
|  |  |  |  | 51 | -36 | 52 | 5.56 | |  | | | | |  | | | | |
|  |  |  |  | 42 | -58 | 49 | 5.36 | |  | | | | |  | | | | |
|  |  |  |  | 50 | -32 | 51 | 5.06 | |  | | | | |  | | | | |
|  |  |  |  | -30 | 14 | 55 | 5.00 | |  | | | | |  | | | | |
|  |  |  |  | -57 | 17 | 30 | 4.12 | |  | | | | |  | | | | |
|  |  |  |  | -32 | 14 | 51 | 4.33 | |  | | | | |  | | | | |
|  |  |  |  | -57 | 17 | 29 | 4.28 | |  | | | | |  | | | | |
|  |  |  |  | -44 | -43 | 41 | 16.93 | | 2 > 0 back | | | | | Control group | | | | |
|  |  |  |  | -42 | -41 | 37 | 11.10 | |  | | | | |  | | | | |
|  |  |  |  | 36 | 6 | 46 | 11.46 | |  | | | | |  | | | | |
|  |  |  |  | -51 | 13 | 32 | 11.23 | |  | | | | |  | | | | |
|  |  |  |  | 2 | 18 | 51 | 9.96 | |  | | | | |  | | | | |
|  |  |  |  | -2 | 16 | 53 | 9.92 | |  | | | | |  | | | | |
|  |  |  |  | 14 | -79 | 11 | 4.87 | |  | | | | |  | | | | |
|  |  |  |  | 26 | -64 | 7 | 3.87 | |  | | | | |  | | | | |
|  |  |  |  | -57 | -51 | -14 | 4.00 | |  | | | | |  | | | | |
|  |  |  |  | -53 | -51 | -16 | 3.83 | |  | | | | |  | | | | |
|  |  |  |  | -55 | -65 | -10 | 3.60 | |  | | | | |  | | | | |
| 12 | Gruber *et al.*, 2010 | MNI | 18 | -36 | 52 | 12 | 4.34 | | Non-articulatory | | | | | Control group | | | | |
|  |  |  |  | 36 | 44 | 28 | 6.00 | |  | | | | |  | | | | |
|  |  |  |  | -44 | -40 | 40 | 4.68 | |  | | | | |  | | | | |
|  |  |  |  | 48 | -40 | 40 | 3.18 | |  | | | | |  | | | | |
|  |  |  |  | 0 | 12 | 52 | 7.74 | |  | | | | |  | | | | |
|  |  |  |  | -28 | -60 | 40 | 6.18 | |  | | | | |  | | | | |
|  |  |  |  | 32 | -56 | 44 | 5.50 | |  | | | | |  | | | | |
|  |  |  |  | -40 | 16 | -4 | 5.64 | |  | | | | |  | | | | |
|  |  |  |  | 32 | 24 | -4 | 6.49 | |  | | | | |  | | | | |
|  |  |  |  | -36 | -56 | -36 | 4.89 | |  | | | | |  | | | | |
|  |  |  |  | 24 | -64 | -28 | 5.16 | |  | | | | |  | | | | |
|  |  |  |  | -16 | -4 | 16 | 4.41 | |  | | | | |  | | | | |
|  |  |  |  | 20 | 4 | 16 | 3.87 | |  | | | | |  | | | | |
|  |  |  |  | -44 | 8 | 24 | 7.63 | |  | | | | |  | | | | |
|  |  |  |  | -48 | -56 | -16 | 4.81 | |  | | | | |  | | | | |
|  |  |  |  | -4 | -24 | -16 | 5.95 | |  | | | | |  | | | | |
| 13 | Honey *et al.*, 2000 | Talairach | 20 | -35 | -53 | 37 | p | | WM > baseline | | | | |  | | | | |
|  |  |  |  | -43 | 17 | 26 | p | |  | | | | |  | | | | |
|  |  |  |  | 35 | 47 | 15 | p | |  | | | | |  | | | | |
|  |  |  |  | 0 | 8 | 48 | p | |  | | | | |  | | | | |
|  |  |  |  | -43 | -6 | 42 | p | |  | | | | |  | | | | |
|  |  |  |  | 32 | -8 | 53 | p | |  | | | | |  | | | | |
|  |  |  |  | -20 | -14 | 53 | p | |  | | | | |  | | | | |
|  |  |  |  | -35 | -72 | -13 | p | |  | | | | |  | | | | |
|  |  |  |  | -35 | -64 | -13 | p | |  | | | | |  | | | | |
|  |  |  |  | 26 | -69 | -18 | p | |  | | | | |  | | | | |
| 14 | Johnson *et al.,* 2006 | Talairach | 18 | -50 | 2 | 44 | 4.9 | | Encoding (color) | | | | | Control group | | | | |
|  |  |  |  | 33 | 47 | 14 | 4.3 | |  | | | | |  | | | | |
|  |  |  |  | 0 | 11 | 52 | 4.1 | |  | | | | |  | | | | |
|  |  |  |  | -27 | -56 | 44 | 3.9 | |  | | | | |  | | | | |
|  |  |  |  | 48 | 17 | -11 | 3.8 | |  | | | | |  | | | | |
|  |  |  |  | -18 | 0 | 8 | 4.3 | |  | | | | |  | | | | |
|  |  |  |  | 36 | 7 | 25 | 3.8 | |  | | | | |  | | | | |
|  |  |  |  | -18 | -3 | -7 | 3.7 | |  | | | | |  | | | | |
|  |  |  |  | 50 | 8 | 44 | 3.4 | |  | | | | |  | | | | |
|  |  |  |  | -42 | 7 | 22 | 5.3 | | Recall (color) | | | | |  | | | | |
|  |  |  |  | -30 | -71 | 45 | 4.7 | |  | | | | |  | | | | |
|  |  |  |  | 0 | 11 | 52 | 4.3 | |  | | | | |  | | | | |
|  |  |  |  | 33 | 50 | 3 | 3.6 | |  | | | | |  | | | | |
|  |  |  |  | -18 | -2 | 8 | 4.0 | |  | | | | |  | | | | |
|  |  |  |  | 50 | 20 | -11 | 3.9 | |  | | | | |  | | | | |
|  |  |  |  | 36 | 7 | 25 | 4.4 | |  | | | | |  | | | | |
|  |  |  |  | -21 | -1 | -10 | 3.8 | |  | | | | |  | | | | |
|  |  |  |  | 50 | 8 | 44 | 3.5 | |  | | | | |  | | | | |
|  |  |  |  | 30 | -2 | -30 | 3.6 | |  | | | | |  | | | | |
|  |  |  |  | -56 | -50 | -13 | 4.0 | |  | | | | |  | | | | |
|  |  |  |  | 0 | -76 | 48 | 3.4 | |  | | | | |  | | | | |
|  |  |  |  | -39 | 55 | 0 | 3.5 | |  | | | | |  | | | | |
| 15 | Karlsgodt *et al.,*2005 | MNI | 13 | 34 | 25 | -2 | 6.42 | | Encoding | | | | |  | | | | |
|  |  |  |  | -41 | 25 | -2 | 4.94 | |  | | | | |  | | | | |
|  |  |  |  | 26 | 12 | -2 | 7.14 | |  | | | | |  | | | | |
|  |  |  |  | -26 | 11 | -4 | 5.93 | |  | | | | |  | | | | |
|  |  |  |  | 58 | -3 | 44 | 7.44 | |  | | | | |  | | | | |
|  |  |  |  | -56 | -1 | 45 | 9.39 | |  | | | | |  | | | | |
|  |  |  |  | 2 | 10 | 47 | 7.65 | |  | | | | |  | | | | |
|  |  |  |  | -8 | 14 | 47 | 9.00 | |  | | | | |  | | | | |
|  |  |  |  | 20 | -90 | -10 | 8.10 | |  | | | | |  | | | | |
|  |  |  |  | -22 | -94 | -9 | 6.36 | |  | | | | |  | | | | |
|  |  |  |  | 45 | -27 | -5 | 5.15 | |  | | | | |  | | | | |
|  |  |  |  | -30 | -66 | 44 | 5.93 | |  | | | | |  | | | | |
|  |  |  |  | 38 | -80 | -17 | 6.66 | |  | | | | |  | | | | |
|  |  |  |  | -43 | -76 | -16 | 8.17 | |  | | | | |  | | | | |
|  |  |  |  | 36 | 29 | -10 | 4.67 | | Maintenance | | | | |  | | | | |
|  |  |  |  | -43 | 4 | 26 | 4.76 | |  | | | | |  | | | | |
|  |  |  |  | 47 | 31 | 23 | 5.03 | |  | | | | |  | | | | |
|  |  |  |  | -57 | -8 | 39 | 5.67 | |  | | | | |  | | | | |
|  |  |  |  | 15 | 10 | 40 | 5.83 | |  | | | | |  | | | | |
|  |  |  |  | -8 | 5 | 50 | 4.67 | |  | | | | |  | | | | |
|  |  |  |  | 41 | -55 | 42 | 4.87 | |  | | | | |  | | | | |
|  |  |  |  | -32 | -62 | 38 | 4.50 | |  | | | | |  | | | | |
|  |  |  |  | 37 | 22 | -1 | 7.65 | | Recall | | | | |  | | | | |
|  |  |  |  | -31 | 21 | -1 | 7.14 | |  | | | | |  | | | | |
|  |  |  |  | -53 | 10 | 26 | 9.12 | |  | | | | |  | | | | |
|  |  |  |  | 36 | 22 | 2 | 7.62 | |  | | | | |  | | | | |
|  |  |  |  | -41 | -6 | 11 | 5.69 | |  | | | | |  | | | | |
|  |  |  |  | 31 | 51 | 10 | 5.36 | |  | | | | |  | | | | |
|  |  |  |  | -32 | 51 | 3 | 6.66 | |  | | | | |  | | | | |
|  |  |  |  | -52 | 3 | 48 | 6.75 | |  | | | | |  | | | | |
|  |  |  |  | 24 | -0 | 7 | 6.10 | |  | | | | |  | | | | |
|  |  |  |  | -20 | 7 | 0 | 5.31 | |  | | | | |  | | | | |
|  |  |  |  | 10 | -10 | 1 | 4.87 | |  | | | | |  | | | | |
|  |  |  |  | -10 | -14 | 4 | 6.60 | |  | | | | |  | | | | |
|  |  |  |  | 20 | -97 | -7 | 11.43 | |  | | | | |  | | | | |
|  |  |  |  | -25 | -94 | 8 | 9.30 | |  | | | | |  | | | | |
|  |  |  |  | -4 | 7 | 48 | 9.21 | |  | | | | |  | | | | |
|  |  |  |  | 49 | 31 | 18 | 9.53 | |  | | | | |  | | | | |
| 16 | Kirschen *et al.*, 2010 | Talairach | 16 | -44 | -60 | -15 | 7.61 | | High load > low load | | | | | Only visual stimuli | | | | |
|  |  |  |  | -33 | -54 | 35 | 8.62 | |  | | | | |  | | | | |
|  |  |  |  | -28 | -74 | 27 | 7.01 | |  | | | | |  | | | | |
|  |  |  |  | -39 | -5 | 54 | 7.61 | |  | | | | |  | | | | |
|  |  |  |  | -46 | 3 | 29 | 7.30 | |  | | | | |  | | | | |
|  |  |  |  | -41 | -37 | -36 | 7.30 | |  | | | | |  | | | | |
|  |  |  |  | 38 | -69 | -7 | 7.93 | |  | | | | |  | | | | |
|  |  |  |  | 33 | -47 | -39 | 8.62 | |  | | | | |  | | | | |
|  |  |  |  | 34 | -60 | -31 | 7.30 | |  | | | | |  | | | | |
|  |  |  |  | 25 | -64 | -32 | 7.01 | |  | | | | |  | | | | |
|  |  |  |  | 39 | -52 | 38 | 5.05 | |  | | | | |  | | | | |
|  |  |  |  | 32 | -50 | 36 | 4.64 | |  | | | | |  | | | | |
|  |  |  |  | 22 | -62 | -53 | 5.05 | |  | | | | |  | | | | |
|  |  |  |  | 41 | -37 | 39 | 5.48 | |  | | | | |  | | | | |
|  |  |  |  | 41 | 4 | 23 | 5.71 | |  | | | | |  | | | | |
| 17 | Knops *et al.,*2006 | Talairach | 16 | -44 | 29 | 28 | 7.55 | | 2 > 1 back(Identity match) | | | | | Only letters | | | | |
|  |  |  |  | -51 | 6 | 44 | 6.43 | |  | | | | |  | | | | |
|  |  |  |  | 28 | 11 | 55 | 5.55 | |  | | | | |  | | | | |
|  |  |  |  | 51 | 32 | 28 | 5.37 | |  | | | | |  | | | | |
|  |  |  |  | 44 | 51 | 12 | 5.17 | |  | | | | |  | | | | |
|  |  |  |  | 28 | 58 | -10 | 6.40 | |  | | | | |  | | | | |
|  |  |  |  | -24 | -64 | 40 | 4.74 | |  | | | | |  | | | | |
|  |  |  |  | 32 | -49 | 36 | 6.98 | |  | | | | |  | | | | |
| 18 | Lim *et al.*, 2008 | Talairach | 12 | -4 | -76 | 36 | p | | 1 back > baseline | | | | | Control group | | | | |
|  |  |  |  | 55 | 15 | 32 | p | |  | | | | |  | | | | |
|  |  |  |  | -40 | -26 | 58 | p | |  | | | | |  | | | | |
|  |  |  |  | -36 | -32 | 28 | p | |  | | | | |  | | | | |
|  |  |  |  | 12 | -16 | 64 | p | |  | | | | |  | | | | |
| 19 | Lythe *et al.*, 2012 | MNI | 20 | 30 | 2 | 58 | 11.20 | | Activations with increasing load | | | | | Control group | | | | |
|  |  |  |  | 36 | -50 | 42 | 8.99 | |  | | | | |  | | | | |
| 20 | Marquand *et al.*, 2008 | Talairach | 20 | 16 | 44 | 52 | p | | 2 > 0 back | | | | | Control Group | | | | |
|  |  |  |  | 2 | 54 | 22 | p | |  | | | | |  | | | | |
|  |  |  |  | -26 | 42 | -14 | p | |  | | | | |  | | | | |
|  |  |  |  | -26 | 48 | -2 | p | |  | | | | |  | | | | |
|  |  |  |  | 36 | 52 | 2 | p | |  | | | | |  | | | | |
|  |  |  |  | 40 | 38 | 42 | p | |  | | | | |  | | | | |
|  |  |  |  | 56 | 16 | 26 | p | |  | | | | |  | | | | |
|  |  |  |  | 54 | 2 | 50 | p | |  | | | | |  | | | | |
|  |  |  |  | 62 | -52 | 16 | p | |  | | | | |  | | | | |
|  |  |  |  | -52 | -4 | -18 | p | |  | | | | |  | | | | |
|  |  |  |  | 56 | 2 | -22 | p | |  | | | | |  | | | | |
|  |  |  |  | 68 | -28 | -6 | p | |  | | | | |  | | | | |
|  |  |  |  | 54 | -60 | 32 | p | |  | | | | |  | | | | |
|  |  |  |  | 0 | -40 | 6 | p | |  | | | | |  | | | | |
|  |  |  |  | 4 | -76 | 56 | p | |  | | | | |  | | | | |
|  |  |  |  | -2 | -100 | 6 | p | |  | | | | |  | | | | |
|  |  |  |  | 4 | -68 | 6 | p | |  | | | | |  | | | | |
|  |  |  |  | 34 | -84 | 38 | p | |  | | | | |  | | | | |
|  |  |  |  | 0 | -82 | -20 | p | |  | | | | |  | | | | |
| 21 | Marvel and Desmond,2010 | Talairach | 16 | 47 | 43 | 6 | 6.20 | | Encoding | | | | | Positive and negative activation | | | | |
|  |  |  |  | -7 | 43 | 32 | 4.84 | |  | | | | |  | | | | |
|  |  |  |  | 13 | 35 | 47 | 5.71 | |  | | | | |  | | | | |
|  |  |  |  | 47 | 26 | -12 | 5.48 | |  | | | | |  | | | | |
|  |  |  |  | -29 | 16 | -7 | 5.05 | |  | | | | |  | | | | |
|  |  |  |  | 17 | 2 | 39 | 4.64 | |  | | | | |  | | | | |
|  |  |  |  | -41 | 0 | -32 | 5.26 | |  | | | | |  | | | | |
|  |  |  |  | -53 | -4 | -8 | 5.95 | |  | | | | |  | | | | |
|  |  |  |  | -40 | -7 | 23 | 7.61 | |  | | | | |  | | | | |
|  |  |  |  | 53 | -15 | 2 | 4.64 | |  | | | | |  | | | | |
|  |  |  |  | -9 | -22 | 36 | 4.64 | |  | | | | |  | | | | |
|  |  |  |  | 34 | -23 | 8 | 6.46 | |  | | | | |  | | | | |
|  |  |  |  | -27 | -24 | -15 | 5.05 | |  | | | | |  | | | | |
|  |  |  |  | -42 | -29 | 53 | 5.48 | |  | | | | |  | | | | |
|  |  |  |  | -48 | -31 | 19 | 5.48 | |  | | | | |  | | | | |
|  |  |  |  | 43 | -32 | 20 | 4.84 | |  | | | | |  | | | | |
|  |  |  |  | -20 | -33 | 40 | 5.48 | |  | | | | |  | | | | |
|  |  |  |  | 58 | -34 | 40 | 4.64 | |  | | | | |  | | | | |
|  |  |  |  | -24 | -42 | 52 | 6.46 | |  | | | | |  | | | | |
|  |  |  |  | 12 | -56 | -36 | 6.46 | |  | | | | |  | | | | |
|  |  |  |  | -53 | -60 | 23 | 5.48 | |  | | | | |  | | | | |
|  |  |  |  | 16 | 34 | -21 | -5.05 | |  | | | | |  | | | | |
|  |  |  |  | -7 | 13 | 5 | -12.15 | |  | | | | |  | | | | |
|  |  |  |  | -51 | 8 | 2 | -4.45 | |  | | | | |  | | | | |
|  |  |  |  | -2 | -5 | 61 | -5.71 | |  | | | | |  | | | | |
|  |  |  |  | -5 | -21 | 9 | -5.26 | |  | | | | |  | | | | |
|  |  |  |  | -7 | -37 | 2 | -4.45 | |  | | | | |  | | | | |
|  |  |  |  | 45 | -58 | -17 | -4.27 | |  | | | | |  | | | | |
|  |  |  |  | 40 | -64 | -36 | -6.46 | |  | | | | |  | | | | |
|  |  |  |  | -46 | -70 | -52 | -4.84 | |  | | | | |  | | | | |
|  |  |  |  | 4 | -72 | 48 | -5.48 | |  | | | | |  | | | | |
|  |  |  |  | -10 | -74 | -20 | -4.64 | |  | | | | |  | | | | |
|  |  |  |  | -9 | -88 | 1 | -5.05 | |  | | | | |  | | | | |
|  |  |  |  | 6 | 68 | 16 | 5.26 | | Maintenance | | | | | Positive and negative activation | | | | |
|  |  |  |  | -9 | 62 | 30 | 6.46 | |  | | | | |  | | | | |
|  |  |  |  | 5 | 57 | 3 | 7.61 | |  | | | | |  | | | | |
|  |  |  |  | -24 | 27 | 57 | 4.45 | |  | | | | |  | | | | |
|  |  |  |  | -21 | 20 | -27 | 6.73 | |  | | | | |  | | | | |
|  |  |  |  | -18 | 5 | 8 | 4.45 | |  | | | | |  | | | | |
|  |  |  |  | 19 | 4 | 3 | 6.73 | |  | | | | |  | | | | |
|  |  |  |  | 60 | -6 | -10 | 5.48 | |  | | | | |  | | | | |
|  |  |  |  | 56 | -8 | 10 | 5.48 | |  | | | | |  | | | | |
|  |  |  |  | 45 | -10 | 46 | 5.26 | |  | | | | |  | | | | |
|  |  |  |  | -42 | -16 | 40 | 4.64 | |  | | | | |  | | | | |
|  |  |  |  | 20 | -17 | -13 | 4.09 | |  | | | | |  | | | | |
|  |  |  |  | -21 | -20 | -21 | 4.84 | |  | | | | |  | | | | |
|  |  |  |  | 0 | -41 | 38 | 4.45 | |  | | | | |  | | | | |
|  |  |  |  | -58 | -54 | -16 | 5.05 | |  | | | | |  | | | | |
|  |  |  |  | 16 | -55 | 66 | 4.45 | |  | | | | |  | | | | |
|  |  |  |  | -57 | -59 | -4 | 4.64 | |  | | | | |  | | | | |
|  |  |  |  | -11 | -61 | 18 | 5.71 | |  | | | | |  | | | | |
|  |  |  |  | -50 | -70 | 26 | 4.84 | |  | | | | |  | | | | |
|  |  |  |  | 32 | -79 | 8 | 4.84 | |  | | | | |  | | | | |
|  |  |  |  | -27 | -84 | 16 | 4.84 | |  | | | | |  | | | | |
|  |  |  |  | -40 | 21 | 40 | -5.05 | |  | | | | |  | | | | |
|  |  |  |  | -29 | 20 | -3 | -8.27 | |  | | | | |  | | | | |
|  |  |  |  | 31 | 19 | -1 | -6.73 | |  | | | | |  | | | | |
|  |  |  |  | 4 | 18 | 53 | -7.30 | |  | | | | |  | | | | |
|  |  |  |  | 62 | 11 | 24 | -5.26 | |  | | | | |  | | | | |
|  |  |  |  | -50 | 10 | 37 | -5.26 | |  | | | | |  | | | | |
|  |  |  |  | -53 | 7 | 17 | -4.64 | |  | | | | |  | | | | |
|  |  |  |  | -5 | 0 | 24 | -5.48 | |  | | | | |  | | | | |
|  |  |  |  | -36 | -9 | 19 | -5.71 | |  | | | | |  | | | | |
|  |  |  |  | -31 | -15 | 62 | -4.09 | |  | | | | |  | | | | |
|  |  |  |  | -52 | -26 | 39 | -8.27 | |  | | | | |  | | | | |
|  |  |  |  | -28 | -32 | 64 | -5.05 | |  | | | | |  | | | | |
|  |  |  |  | 12 | -56 | -14 | -7.30 | |  | | | | |  | | | | |
|  |  |  |  | -6 | -64 | -40 | -6.20 | |  | | | | |  | | | | |
|  |  |  |  | 14 | -70 | -46 | -5.71 | |  | | | | |  | | | | |
|  |  |  |  | 31 | 24 | 7 | 5.26 | | Recall | | | | | Positive and negative activation | | | | |
|  |  |  |  | -29 | 22 | 11 | 5.26 | |  | | | | |  | | | | |
|  |  |  |  | -40 | 19 | 23 | 5.71 | |  | | | | |  | | | | |
|  |  |  |  | -5 | 5 | 53 | 5.95 | |  | | | | |  | | | | |
|  |  |  |  | 12 | -57 | -40 | 5.48 | |  | | | | |  | | | | |
|  |  |  |  | 17 | 38 | 41 | -4.27 | |  | | | | |  | | | | |
|  |  |  |  | 8 | 34 | 56 | -4.27 | |  | | | | |  | | | | |
|  |  |  |  | 42 | 29 | 11 | -4.84 | |  | | | | |  | | | | |
|  |  |  |  | 23 | 6 | 0 | -5.05 | |  | | | | |  | | | | |
|  |  |  |  | -46 | -12 | 35 | -4.27 | |  | | | | |  | | | | |
|  |  |  |  | 20 | -15 | -17 | -4.27 | |  | | | | |  | | | | |
|  |  |  |  | -62 | -41 | 1 | -5.05 | |  | | | | |  | | | | |
|  |  |  |  | 43 | -50 | 29 | -4.27 | |  | | | | |  | | | | |
|  |  |  |  | -52 | -53 | 33 | -4.45 | |  | | | | |  | | | | |
|  |  |  |  | 43 | -63 | 31 | -4.84 | |  | | | | |  | | | | |
| 22 | McMillan *et al.*, 2007 | Talairach | 16 | 46 | 7 | 31 | 0.99 | | 2 > 0 back | | | | | Color identification condition | | | | |
|  |  |  |  | 33 | 45 | 33 | 0.58 | |  | | | | |  | | | | |
|  |  |  |  | 35 | 24 | 4 | 0.51 | |  | | | | |  | | | | |
|  |  |  |  | -36 | 1 | 53 | 0.84 | |  | | | | |  | | | | |
|  |  |  |  | 0 | 3 | 54 | 1.00 | |  | | | | |  | | | | |
|  |  |  |  | 29 | -10 | 59 | 0.75 | |  | | | | |  | | | | |
|  |  |  |  | 41 | 44 | 12 | 0.58 | |  | | | | |  | | | | |
|  |  |  |  | 9 | -76 | 29 | 1.02 | |  | | | | |  | | | | |
|  |  |  |  | -1 | -84 | -16 | 0.62 | |  | | | | |  | | | | |
|  |  |  |  | -38 | -58 | 30 | 0.60 | |  | | | | |  | | | | |
|  |  |  |  | 45 | 8 | 37 | 0.84 | | 2 > 0 back | | | | | Identity condition | | | | |
|  |  |  |  | 32 | 25 | -2 | 0.33 | |  | | | | |  | | | | |
|  |  |  |  | 0 | 4 | 54 | 0.90 | |  | | | | |  | | | | |
|  |  |  |  | -29 | -2 | 61 | 0.89 | |  | | | | |  | | | | |
|  |  |  |  | 30 | -7 | 59 | 0.65 | |  | | | | |  | | | | |
|  |  |  |  | -42 | 14 | 46 | 0.53 | |  | | | | |  | | | | |
|  |  |  |  | 32 | 45 | 24 | 0.48 | |  | | | | |  | | | | |
|  |  |  |  | -2 | -80 | 29 | 1.04 | |  | | | | |  | | | | |
|  |  |  |  | -34 | -74 | 24 | 0.76 | |  | | | | |  | | | | |
|  |  |  |  | -2 | -90 | -16 | 0.58 | |  | | | | |  | | | | |
|  |  |  |  | 32 | -74 | 30 | 0.58 | |  | | | | |  | | | | |
| 23 | McNab *et al.,* 2008 | MNI | 11 | -42 | 3 | 30 | 11.08 | | WM > control | | | | |  | | | | |
|  |  |  |  | -3 | -3 | 63 | 10.35 | |  | | | | |  | | | | |
|  |  |  |  | -42 | -6 | 51 | 9.89 | |  | | | | |  | | | | |
|  |  |  |  | -30 | -60 | 42 | 8.46 | |  | | | | |  | | | | |
|  |  |  |  | -27 | -69 | 45 | 8.10 | |  | | | | |  | | | | |
|  |  |  |  | -42 | -45 | 42 | 7.88 | |  | | | | |  | | | | |
|  |  |  |  | 36 | 21 | -6 | 7.65 | |  | | | | |  | | | | |
|  |  |  |  | 30 | -69 | 48 | 7.60 | |  | | | | |  | | | | |
|  |  |  |  | 39 | -48 | 48 | 6.89 | |  | | | | |  | | | | |
|  |  |  |  | 33 | -66 | 36 | 6.47 | |  | | | | |  | | | | |
|  |  |  |  | 45 | 3 | 30 | 5.66 | |  | | | | |  | | | | |
|  |  |  |  | 51 | 24 | 30 | 5.29 | |  | | | | |  | | | | |
|  |  |  |  | 45 | 42 | 30 | 4.58 | |  | | | | |  | | | | |
|  |  |  |  | 33 | -3 | 66 | 5.26 | |  | | | | |  | | | | |
|  |  |  |  | 27 | -6 | 60 | 4.97 | |  | | | | |  | | | | |
|  |  |  |  | -48 | -51 | 6 | 5.00 | |  | | | | |  | | | | |
|  |  |  |  | -15 | -3 | 15 | 4.65 | |  | | | | |  | | | | |
|  |  |  |  | -15 | 9 | 3 | 4.22 | |  | | | | |  | | | | |
|  |  |  |  | -3 | -18 | 15 | 4.22 | |  | | | | |  | | | | |
| 24 | Meisenzahl *et al.*,2006 | Talairach | 12 | -49 | 21 | -9 | 6.02 | | 2 back > baseline | | | | | Control group | | | | |
|  |  |  |  | -46 | -42 | 38 | 8.98 | |  | | | | |  | | | | |
|  |  |  |  | -42 | -44 | 48 | 5.65 | |  | | | | |  | | | | |
|  |  |  |  | 48 | 11 | 23 | 8.34 | |  | | | | |  | | | | |
|  |  |  |  | 51 | 22 | 15 | 4.09 | |  | | | | |  | | | | |
|  |  |  |  | -12 | -11 | 10 | 8.15 | |  | | | | |  | | | | |
|  |  |  |  | -10 | 2 | 9 | 7.52 | |  | | | | |  | | | | |
|  |  |  |  | 6 | -19 | 3 | 6.91 | |  | | | | |  | | | | |
|  |  |  |  | -40 | 1 | 29 | 7.74 | |  | | | | |  | | | | |
|  |  |  |  | 34 | 21 | -16 | 7.20 | |  | | | | |  | | | | |
|  |  |  |  | -46 | 23 | 26 | 7.00 | |  | | | | |  | | | | |
|  |  |  |  | 46 | -41 | 41 | 5.96 | |  | | | | |  | | | | |
|  |  |  |  | -63 | -40 | 24 | 5.67 | |  | | | | |  | | | | |
|  |  |  |  | 32 | -60 | 49 | 5.63 | |  | | | | |  | | | | |
|  |  |  |  | -36 | 47 | 7 | 5.54 | |  | | | | |  | | | | |
|  |  |  |  | -34 | 42 | 15 | 5.09 | |  | | | | |  | | | | |
|  |  |  |  | -42 | 10 | 40 | 4.58 | |  | | | | |  | | | | |
|  |  |  |  | -2 | 36 | 18 | 4.50 | |  | | | | |  | | | | |
|  |  |  |  | -26 | 21 | -6 | 14.19 | |  | | | | |  | | | | |
|  |  |  |  | 2 | 32 | 28 | 7.89 | |  | | | | |  | | | | |
| 25 | Monks *et al.*, 2004 | Talairach | 12 | 15 | 0 | 42 | p | | 2 back > baseline | | | | | Control group | | | | |
|  |  |  |  | -25 | -60 | 37 | p | |  | | | | |  | | | | |
|  |  |  |  | -11 | -10 | 48 | p | |  | | | | |  | | | | |
|  |  |  |  | -50 | -17 | 37 | p | |  | | | | |  | | | | |
|  |  |  |  | 53 | -7 | 26 | p | |  | | | | |  | | | | |
|  |  |  |  | 36 | -56 | 31 | p | |  | | | | |  | | | | |
|  |  |  |  | 43 | 10 | -2 | p | |  | | | | |  | | | | |
|  |  |  |  | -43 | 10 | -2 | p | |  | | | | |  | | | | |
|  |  |  |  | -4 | -46 | 20 | p | |  | | | | |  | | | | |
|  |  |  |  | -40 | -23 | 53 | p | |  | | | | |  | | | | |
|  |  |  |  | -36 | 10 | 26 | p | |  | | | | |  | | | | |
|  |  |  |  | 36 | -67 | 15 | p | |  | | | | |  | | | | |
|  |  |  |  | 11 | 50 | -13 | p | |  | | | | |  | | | | |
|  |  |  |  | -40 | 0 | 37 | p | |  | | | | |  | | | | |
|  |  |  |  | 4 | 7 | 48 | p | |  | | | | |  | | | | |
|  |  |  |  | 11 | 39 | -29 | p | |  | | | | |  | | | | |
|  |  |  |  | 50 | -10 | 37 | p | |  | | | | |  | | | | |
|  |  |  |  | 3 | -80 | -24 | p | | Sternberg task | | | | | Control group | | | | |
|  |  |  |  | 32 | -72 | 28 | p | |  | | | | |  | | | | |
|  |  |  |  | 4 | -75 | 1 | p | |  | | | | |  | | | | |
|  |  |  |  | -47 | -22 | 40 | p | |  | | | | |  | | | | |
|  |  |  |  | -42 | -70 | 28 | p | |  | | | | |  | | | | |
|  |  |  |  | 56 | -35 | 28 | p | |  | | | | |  | | | | |
|  |  |  |  | -19 | -82 | -32 | p | |  | | | | |  | | | | |
|  |  |  |  | 37 | -3 | 32 | p | |  | | | | |  | | | | |
|  |  |  |  | 41 | 16 | 32 | p | |  | | | | |  | | | | |
|  |  |  |  | -41 | 0 | 28 | p | |  | | | | |  | | | | |
|  |  |  |  | -44 | -65 | 20 | p | |  | | | | |  | | | | |
|  |  |  |  | 1 | -63 | 16 | p | |  | | | | |  | | | | |
|  |  |  |  | -1 | 2 | 45 | p | |  | | | | |  | | | | |
|  |  |  |  | 50 | 10 | 4 | p | |  | | | | |  | | | | |
|  |  |  |  | -33 | -72 | 35 | p | |  | | | | |  | | | | |
|  |  |  |  | -27 | -9 | 1 | p | |  | | | | |  | | | | |
|  |  |  |  | 40 | -3 | -36 | p | |  | | | | |  | | | | |
| 26 | Mu *et al.*, 2008 | MNI | 33 | -48 | 2 | 24 | 9.47 | | Task > control | | | | |  | | | | |
|  |  |  |  | -30 | -76 | 20 | 10.47 | |  | | | | |  | | | | |
|  |  |  |  | -44 | 36 | 6 | 7.63 | |  | | | | |  | | | | |
|  |  |  |  | -2 | -14 | 58 | 8.43 | |  | | | | |  | | | | |
|  |  |  |  | 34 | 14 | -2 | 8.34 | |  | | | | |  | | | | |
|  |  |  |  | 30 | -78 | 22 | 7.68 | |  | | | | |  | | | | |
| 27 | Narayanan *et al.,* 2005 | MNI | 12 | -50 | 2 | 32 | 5.78 | | Encoding | | | | |  | | | | |
|  |  |  |  | -34 | 26 | 0 | 7.26 | |  | | | | |  | | | | |
|  |  |  |  | -10 | -18 | 44 | 4.86 | |  | | | | |  | | | | |
|  |  |  |  | -44 | -78 | 2 | 5.28 | |  | | | | |  | | | | |
|  |  |  |  | 42 | -70 | 2 | 6.62 | |  | | | | |  | | | | |
|  |  |  |  | -12 | -86 | -6 | 8.37 | |  | | | | |  | | | | |
|  |  |  |  | 8 | -88 | -6 | 5.38 | |  | | | | |  | | | | |
|  |  |  |  | -28 | -84 | -2 | 7.43 | |  | | | | |  | | | | |
|  |  |  |  | 42 | -66 | -2 | 8.23 | |  | | | | |  | | | | |
|  |  |  |  | -10 | -26 | -2 | 8.68 | |  | | | | |  | | | | |
|  |  |  |  | -20 | 10 | 2 | 5.15 | |  | | | | |  | | | | |
|  |  |  |  | -32 | 26 | -2 | 7.19 | |  | | | | |  | | | | |
|  |  |  |  | -58 | 4 | 32 | 6.91 | | Maintenance | | | | |  | | | | |
|  |  |  |  | -6 | 24 | 46 | 6.59 | |  | | | | |  | | | | |
|  |  |  |  | -58 | 6 | 30 | 6.00 | |  | | | | |  | | | | |
|  |  |  |  | -50 | 10 | 18 | 4.87 | |  | | | | |  | | | | |
|  |  |  |  | -36 | 26 | 2 | 5.21 | |  | | | | |  | | | | |
|  |  |  |  | -4 | 22 | 46 | 6.26 | |  | | | | |  | | | | |
|  |  |  |  | -34 | -50 | 52 | 5.97 | |  | | | | |  | | | | |
|  |  |  |  | -12 | 10 | 6 | 5.32 | |  | | | | |  | | | | |
|  |  |  |  | -46 | 6 | 38 | 5.95 | | Recall | | | | |  | | | | |
|  |  |  |  | -50 | 22 | 32 | 6.99 | |  | | | | |  | | | | |
|  |  |  |  | -46 | 2 | 40 | 6.46 | |  | | | | |  | | | | |
|  |  |  |  | -50 | 30 | 8 | 5.00 | |  | | | | |  | | | | |
|  |  |  |  | -34 | 24 | 2 | 10.73 | |  | | | | |  | | | | |
|  |  |  |  | -40 | 16 | 6 | 6.94 | |  | | | | |  | | | | |
|  |  |  |  | 40 | 16 | 2 | 7.52 | |  | | | | |  | | | | |
|  |  |  |  | -8 | 6 | 52 | 5.44 | |  | | | | |  | | | | |
|  |  |  |  | -46 | 0 | 40 | 6.61 | |  | | | | |  | | | | |
|  |  |  |  | -38 | -48 | 54 | 5.44 | |  | | | | |  | | | | |
|  |  |  |  | 40 | 18 | 4 | 7.69 | |  | | | | |  | | | | |
|  |  |  |  | -44 | -36 | 48 | 5.84 | |  | | | | |  | | | | |
|  |  |  |  | -44 | -34 | 48 | 5.72 | |  | | | | |  | | | | |
|  |  |  |  | -4 | -28 | 0 | 7.67 | |  | | | | |  | | | | |
|  |  |  |  | -34 | 24 | -2 | 8.35 | |  | | | | |  | | | | |
|  |  |  |  | 36 | 18 | 4 | 6.03 | |  | | | | |  | | | | |
| 28 | Norbury *et al.,* 2014 | MNI | 15 | 28 | 4 | 56 | 8.65 | | Tasks > control | | | | |  | | | | |
|  |  |  |  | 42 | -38 | 34 | 6.14 | |  | | | | |  | | | | |
|  |  |  |  | 38 | -56 | -38 | 6.46 | |  | | | | |  | | | | |
|  |  |  |  | -30 | -48 | 36 | 4.56 | |  | | | | |  | | | | |
|  |  |  |  | -30 | -56 | -38 | 5.38 | |  | | | | |  | | | | |
|  |  |  |  | -4 | -38 | -24 | 4.03 | |  | | | | |  | | | | |
| 29 | Ragland *et al.*, 2002 | Talairach | 11 | 36 | 22 | 16 | 9.47 | | 1 back > 0 back | | | | | Only letters | | | | |
|  |  |  |  | 36 | 18 | 28 | 11.81 | |  | | | | |  | | | | |
|  |  |  |  | 28 | -6 | 36 | 8.08 | |  | | | | |  | | | | |
|  |  |  |  | 36 | -42 | 36 | 12.50 | |  | | | | |  | | | | |
|  |  |  |  | -32 | -50 | 40 | 8.08 | |  | | | | |  | | | | |
|  |  |  |  | -44 | -38 | 40 | 8.51 | |  | | | | |  | | | | |
|  |  |  |  | 36 | 18 | 0 | 8.51 | | 2 back > 0 back | | | | | Only letters | | | | |
|  |  |  |  | 48 | -50 | 4 | 10.00 | |  | | | | |  | | | | |
|  |  |  |  | 36 | 54 | 4 | 7.67 | |  | | | | |  | | | | |
|  |  |  |  | 44 | 34 | 28 | 13.23 | |  | | | | |  | | | | |
|  |  |  |  | -48 | 14 | 8 | 7.67 | |  | | | | |  | | | | |
|  |  |  |  | 44 | -42 | 40 | 11.81 | |  | | | | |  | | | | |
|  |  |  |  | -52 | -42 | 40 | 10.00 | |  | | | | |  | | | | |
| 30 | Ravizza *et al.,*2004 | Talairach | 10 | 2 | 19 | 48 | p | | 3 back > 0 back | | | | | 1.5 T | | | | |
|  |  |  |  | -29 | -3 | 56 | p | |  | | | | |  | | | | |
|  |  |  |  | 31 | 5 | 58 | p | |  | | | | |  | | | | |
|  |  |  |  | -39 | 23 | 36 | p | |  | | | | |  | | | | |
|  |  |  |  | -33 | 46 | 28 | p | |  | | | | |  | | | | |
|  |  |  |  | 46 | 8 | 39 | p | |  | | | | |  | | | | |
|  |  |  |  | 40 | 35 | 33 | p | |  | | | | |  | | | | |
|  |  |  |  | -46 | 7 | 34 | p | |  | | | | |  | | | | |
|  |  |  |  | 34 | 23 | 7 | p | |  | | | | |  | | | | |
|  |  |  |  | -42 | -47 | 38 | p | |  | | | | |  | | | | |
|  |  |  |  | 48 | -51 | 40 | p | |  | | | | |  | | | | |
|  |  | Talairach | 11 | 6 | 17 | 46 | p | | 3 back > 0 back | | | | | 3 T | | | | |
|  |  |  |  | -23 | -2 | 50 | p | |  | | | | |  | | | | |
|  |  |  |  | 27 | -1 | 45 | p | |  | | | | |  | | | | |
|  |  |  |  | 47 | -2 | 37 | p | |  | | | | |  | | | | |
|  |  |  |  | -30 | 47 | 17 | p | |  | | | | |  | | | | |
|  |  |  |  | 43 | 19 | 40 | p | |  | | | | |  | | | | |
|  |  |  |  | 34 | 32 | 22 | p | |  | | | | |  | | | | |
|  |  |  |  | -41 | 17 | 36 | p | |  | | | | |  | | | | |
|  |  |  |  | 42 | -71 | 43 | p | |  | | | | |  | | | | |
|  |  |  |  | -47 | -55 | 44 | p | |  | | | | |  | | | | |
|  |  |  |  | 46 | -48 | 42 | p | |  | | | | |  | | | | |
|  |  |  |  | -23 | -58 | -29 | p | |  | | | | |  | | | | |
|  |  |  |  | -31 | -41 | -31 | p | |  | | | | |  | | | | |
|  |  |  |  | 30 | -61 | -28 | p | |  | | | | |  | | | | |
| 31 | Scheuerecker *et al.*, 2008 | MNI | 23 | -32 | 20 | -4 | 7.63 | | 2 back > 0 back | | | | | Control group | | | | |
|  |  |  |  | 0 | -24 | 8 | 6.27 | |  | | | | |  | | | | |
|  |  |  |  | -54 | -44 | 40 | 6.13 | |  | | | | |  | | | | |
|  |  |  |  | 34 | -64 | 48 | 5.46 | |  | | | | |  | | | | |
|  |  |  |  | 50 | 20 | 22 | 5.15 | |  | | | | |  | | | | |
|  |  |  |  | 32 | 22 | -4 | 4.81 | |  | | | | |  | | | | |
|  |  |  |  | -36 | 54 | 4 | 4.67 | |  | | | | |  | | | | |
|  |  |  |  | 36 | -62 | -22 | 4.50 | |  | | | | |  | | | | |
| 32 | Schloesser *et al.*, 2008 | Talairach | 41 | -6 | -67 | 51 | 9.58 | | Alphabatize > forward | | | | | Control group | | | | |
|  |  |  |  | -30 | -60 | 42 | 5.94 | |  | | | | |  | | | | |
|  |  |  |  | -38 | -41 | 39 | 5.97 | |  | | | | |  | | | | |
|  |  |  |  | 30 | 14 | 55 | 7.14 | |  | | | | |  | | | | |
|  |  |  |  | -42 | 10 | 38 | 6.47 | |  | | | | |  | | | | |
|  |  |  |  | 48 | 36 | 15 | 6.94 | |  | | | | |  | | | | |
|  |  |  |  | 2 | 23 | 39 | 7.27 | |  | | | | |  | | | | |
|  |  |  |  | 38 | -65 | -24 | 8.99 | |  | | | | |  | | | | |
|  |  |  |  | -32 | -60 | -29 | 7.84 | |  | | | | |  | | | | |
|  |  |  |  | 4 | -22 | 9 | 6.18 | |  | | | | |  | | | | |
| 33 | Schmidt *et al.*, 2009 | Talairach | 25 | 26 | 12 | 54 | 8.37 | | All Tasks (1,2,3) > 0 back | | | | | Only male participants | | | | |
|  |  |  |  | -6 | 22 | 46 | 6.93 | |  | | | | |  | | | | |
|  |  |  |  | 42 | 36 | 32 | 6.40 | |  | | | | |  | | | | |
|  |  |  |  | 32 | 64 | 8 | 5.55 | |  | | | | |  | | | | |
|  |  |  |  | -34 | 22 | -4 | 5.45 | |  | | | | |  | | | | |
|  |  |  |  | 14 | -68 | 52 | 7.39 | |  | | | | |  | | | | |
|  |  |  |  | -28 | 0 | 58 | 6.77 | |  | | | | |  | | | | |
|  |  |  |  | -48 | -48 | 50 | 6.59 | |  | | | | |  | | | | |
|  |  |  | 21 | 34 | 4 | 56 | 7.20 | | All Tasks (1,2,3) > 0 back | | | | | Only female participants | | | | |
|  |  |  |  | -4 | 14 | 54 | 7.02 | |  | | | | |  | | | | |
|  |  |  |  | 54 | 22 | 30 | 5.70 | |  | | | | |  | | | | |
|  |  |  |  | -56 | 20 | 32 | 9.27 | |  | | | | |  | | | | |
|  |  |  |  | -41 | 32 | -8 | 6.41 | |  | | | | |  | | | | |
|  |  |  |  | 44 | -54 | 58 | 7.03 | |  | | | | |  | | | | |
| 34 | Seo *et al.,*2012 | MNI | 22 | -42 | 9 | 30 | 16.13 | | 2 back > 0 back | | | | | Control group | | | | |
|  |  |  |  | 30 | 9 | 57 | 15.10 | |  | | | | |  | | | | |
|  |  |  |  | -33 | 24 | -6 | 9.91 | |  | | | | |  | | | | |
|  |  |  |  | 33 | 26 | -6 | 9.20 | |  | | | | |  | | | | |
|  |  |  |  | -6 | 24 | 45 | 10.91 | |  | | | | |  | | | | |
|  |  |  |  | 9 | 27 | 45 | 11.82 | |  | | | | |  | | | | |
|  |  |  |  | -3 | 15 | 54 | 12.56 | |  | | | | |  | | | | |
|  |  |  |  | 6 | 18 | 54 | 8.70 | |  | | | | |  | | | | |
|  |  |  |  | -15 | 3 | 0 | 12.18 | |  | | | | |  | | | | |
|  |  |  |  | 15 | 3 | 0 | 9.10 | |  | | | | |  | | | | |
|  |  |  |  | -12 | 9 | 0 | 7.27 | |  | | | | |  | | | | |
|  |  |  |  | 15 | 9 | 6 | 7.42 | |  | | | | |  | | | | |
|  |  |  |  | -54 | -51 | -15 | 4.98 | |  | | | | |  | | | | |
|  |  |  |  | -27 | -66 | 45 | 9.77 | |  | | | | |  | | | | |
|  |  |  |  | 39 | -66 | 51 | 7.48 | |  | | | | |  | | | | |
|  |  |  |  | -39 | -60 | 48 | 10.66 | |  | | | | |  | | | | |
|  |  |  |  | 42 | -45 | 42 | 9.68 | |  | | | | |  | | | | |
|  |  |  |  | -9 | -78 | -34 | 6.39 | |  | | | | |  | | | | |
|  |  |  |  | 35 | -63 | -39 | 11.07 | |  | | | | |  | | | | |
| 35 | Valera *et al.*,2005 | Talairach | 20 | -33 | 27 | -6 | 14.06 | | 2 back > 0 back | | | | | Control group | | | | |
|  |  |  |  | -27 | -69 | 42 | 9.33 | |  | | | | |  | | | | |
|  |  |  |  | 9 | -75 | -24 | 8.77 | |  | | | | |  | | | | |
|  |  |  |  | 15 | -81 | 12 | 6.13 | |  | | | | |  | | | | |
|  |  |  |  | 57 | -54 | -18 | 5.77 | |  | | | | |  | | | | |
| 36 | Veltman *et al.*, 2003 | Talairach | 21 | -36 | 48 | 21 | 8.59 | | n-back | | | | | Modulated by load | | | | |
|  |  |  |  | -48 | 24 | 30 | 14.95 | |  | | | | |  | | | | |
|  |  |  |  | -45 | 36 | 27 | 13.33 | |  | | | | |  | | | | |
|  |  |  |  | -51 | 12 | 9 | 15.54 | |  | | | | |  | | | | |
|  |  |  |  | 54 | 15 | 36 | 12.36 | |  | | | | |  | | | | |
|  |  |  |  | 45 | 36 | 36 | 13.84 | |  | | | | |  | | | | |
|  |  |  |  | 54 | 12 | 18 | 12.36 | |  | | | | |  | | | | |
|  |  |  |  | -45 | -39 | 51 | 22.31 | |  | | | | |  | | | | |
|  |  |  |  | -45 | -54 | -15 | 7.72 | |  | | | | |  | | | | |
|  |  |  |  | 9 | 12 | 63 | 20.59 | |  | | | | |  | | | | |
|  |  |  |  | 39 | -57 | -42 | 12.83 | |  | | | | |  | | | | |
|  |  |  |  | -51 | 12 | 36 | 9.23 | | Sternberg | | | | | Modulated by load | | | | |
|  |  |  |  | -48 | 24 | 27 | 8.29 | |  | | | | |  | | | | |
|  |  |  |  | -51 | 12 | 3 | 8.00 | |  | | | | |  | | | | |
|  |  |  |  | 54 | 12 | 36 | 6.95 | |  | | | | |  | | | | |
|  |  |  |  | 45 | 33 | 33 | 7.46 | |  | | | | |  | | | | |
|  |  |  |  | -45 | -42 | 54 | 6.71 | |  | | | | |  | | | | |
|  |  |  |  | 6 | 18 | 60 | 8.59 | |  | | | | |  | | | | |
|  |  |  |  | 21 | -57 | -42 | 6.47 | |  | | | | |  | | | | |
| 37 | Walter et al., 2003 | Talairach | 13 | 30 | 3 | 51 | p | | 2 > 0 back (Color identification) | | | | | Control group | | | | |
|  |  |  |  | -27 | 0 | 57 | p | |  | | | | |  | | | | |
|  |  |  |  | 3 | 21 | 45 | p | |  | | | | |  | | | | |
|  |  |  |  | 36 | 21 | -3 | p | |  | | | | |  | | | | |
|  |  |  |  | -33 | 18 | -3 | p | |  | | | | |  | | | | |
|  |  |  |  | 45 | 9 | 33 | p | |  | | | | |  | | | | |
|  |  |  |  | -48 | 12 | 27 | p | |  | | | | |  | | | | |
|  |  |  |  | 39 | 39 | 27 | p | |  | | | | |  | | | | |
|  |  |  |  | -45 | 27 | 27 | p | |  | | | | |  | | | | |
|  |  |  |  | 36 | 54 | 0 | p | |  | | | | |  | | | | |
|  |  |  |  | -45 | 48 | 6 | p | |  | | | | |  | | | | |
|  |  |  |  | 24 | -66 | 57 | p | |  | | | | |  | | | | |
|  |  |  |  | -18 | -66 | 57 | p | |  | | | | |  | | | | |
|  |  |  |  | 36 | -48 | 42 | p | |  | | | | |  | | | | |
|  |  |  |  | -30 | -51 | 42 | p | |  | | | | |  | | | | |
|  |  |  |  | -42 | 3 | 45 | p | |  | | | | |  | | | | |
|  |  |  |  | -6 | -24 | 6 | 7.20 | |  | | | | |  | | | | |
|  |  |  |  | 36 | 9 | 57 | p | | 2 > 0 back (Identity match) | | | | | Control group | | | | |
|  |  |  |  | -30 | 0 | 57 | p | |  | | | | |  | | | | |
|  |  |  |  | 3 | 21 | 45 | p | |  | | | | |  | | | | |
|  |  |  |  | 36 | 21 | -3 | p | |  | | | | |  | | | | |
|  |  |  |  | -33 | 18 | 0 | p | |  | | | | |  | | | | |
|  |  |  |  | 45 | 9 | 30 | p | |  | | | | |  | | | | |
|  |  |  |  | -48 | 9 | 30 | p | |  | | | | |  | | | | |
|  |  |  |  | 39 | 39 | 27 | p | |  | | | | |  | | | | |
|  |  |  |  | -45 | 21 | 27 | p | |  | | | | |  | | | | |
|  |  |  |  | 36 | 51 | 9 | p | |  | | | | |  | | | | |
|  |  |  |  | -39 | 51 | 12 | p | |  | | | | |  | | | | |
|  |  |  |  | 30 | -63 | 54 | p | |  | | | | |  | | | | |
|  |  |  |  | -24 | -66 | 51 | p | |  | | | | |  | | | | |
|  |  |  |  | 39 | -48 | 39 | p | |  | | | | |  | | | | |
|  |  |  |  | -33 | -48 | 39 | p | |  | | | | |  | | | | |
|  |  |  |  | -42 | 3 | 45 | p | |  | | | | |  | | | | |
|  |  |  |  | 9 | -24 | -9 | 16.89 | |  | | | | |  | | | | |
|  |  |  |  | -6 | -24 | -6 | 20.85 | |  | | | | |  | | | | |
| 38 | Walter et al., 2007 | Talairach | 13 | -36 | -81 | -12 | 10.88 | | L1 > control | | | | | Control group | | | | |
|  |  |  |  | 42 | -69 | -24 | 9.65 | |  | | | | |  | | | | |
|  |  |  |  | -42 | -42 | 45 | 8.34 | |  | | | | |  | | | | |
|  |  |  |  | -51 | 3 | 36 | 8.11 | |  | | | | |  | | | | |
|  |  |  |  | -24 | -69 | 54 | 7.67 | |  | | | | |  | | | | |
|  |  |  |  | 33 | -63 | 54 | 7.02 | |  | | | | |  | | | | |
|  |  |  |  | -51 | 3 | 36 | p | | L2 > control | | | | | Control group | | | | |
|  |  |  |  | 21 | -66 | 60 | p | |  | | | | |  | | | | |
|  |  |  |  | 30 | -66 | -27 | 27.35 | |  | | | | |  | | | | |
|  |  |  |  | 30 | -3 | 66 | 18.33 | |  | | | | |  | | | | |
|  |  |  |  | -33 | -69 | -24 | 15.15 | |  | | | | |  | | | | |
|  |  |  |  | -39 | 24 | 0 | 12.51 | |  | | | | |  | | | | |
|  |  |  |  | 33 | 24 | 3 | 10.57 | |  | | | | |  | | | | |
|  |  |  |  | -21 | 0 | 9 | 9.77 | |  | | | | |  | | | | |
|  |  |  |  | 42 | 36 | 24 | 8.75 | |  | | | | |  | | | | |
|  |  |  |  | -42 | 24 | 27 | 7.70 | |  | | | | |  | | | | |
|  |  |  |  | 12 | -75 | -21 | 7.31 | |  | | | | |  | | | | |
|  |  |  |  | -18 | -69 | 57 | p | | L3 > control | | | | | Control group | | | | |
|  |  |  |  | -51 | 3 | 36 | p | |  | | | | |  | | | | |
|  |  |  |  | 24 | -66 | 60 | p | |  | | | | |  | | | | |
|  |  |  |  | 39 | 39 | 24 | 18.76 | |  | | | | |  | | | | |
|  |  |  |  | 54 | 12 | 30 | 12.14 | |  | | | | |  | | | | |
|  |  |  |  | 24 | -63 | 6 | 11.40 | |  | | | | |  | | | | |
|  |  |  |  | -39 | -42 | -36 | 7.55 | |  | | | | |  | | | | |
|  |  |  |  | -66 | -15 | 18 | 7.17 | |  | | | | |  | | | | |
|  |  |  |  | -33 | 51 | 18 | 7.14 | |  | | | | |  | | | | |
|  |  |  |  | -33 | -39 | -42 | 7.11 | |  | | | | |  | | | | |
| 39 | Wishart *et al.,* 2006 | MNI | 22 | 50 | -48 | 44 | 9.09 | | 2 > 0 back | | | | | Control group | | | | |
|  |  |  |  | -28 | -70 | 40 | 8.08 | |  | | | | |  | | | | |
|  |  |  |  | -28 | -78 | 24 | 5.56 | |  | | | | |  | | | | |
|  |  |  |  | 52 | 16 | 34 | 8.48 | |  | | | | |  | | | | |
|  |  |  |  | 44 | 8 | 32 | 8.36 | |  | | | | |  | | | | |
|  |  |  |  | 32 | 2 | 58 | 7.06 | |  | | | | |  | | | | |
|  |  |  |  | -24 | 4 | 60 | 7.28 | |  | | | | |  | | | | |
|  |  |  |  | -46 | 16 | 26 | 6.04 | |  | | | | |  | | | | |
|  |  |  |  | -40 | 4 | 28 | 4.43 | |  | | | | |  | | | | |
|  |  |  |  | 2 | -4 | 12 | 4.05 | |  | | | | |  | | | | |
|  |  |  |  | 10 | -20 | 18 | 2.94 | |  | | | | |  | | | | |
|  |  |  |  | 44 | 58 | 14 | 4.27 | |  | | | | |  | | | | |
|  |  |  |  | 48 | 54 | 0 | 3.76 | |  | | | | |  | | | | |
|  |  |  |  | 38 | 58 | 4 | 3.3 | |  | | | | |  | | | | |
| 40 | Wolf *et al.,* 2006 | Talairach | 15 | 42 | 15 | -3 | 3.74 | | L2 > L1 | | | | |  | | | | |
|  |  |  |  | -18 | 33 | 27 | 4.03 | |  | | | | |  | | | | |
|  |  |  |  | -42 | 18 | 27 | 5.01 | |  | | | | |  | | | | |
|  |  |  |  | 42 | 30 | 33 | 4.66 | |  | | | | |  | | | | |
|  |  |  |  | -39 | 54 | 21 | 5.76 | |  | | | | |  | | | | |
|  |  |  |  | -45 | 18 | 3 | 4.00 | | L3 > L2 | | | | |  | | | | |
|  |  |  |  | 45 | 45 | 27 | 5.62 | |  | | | | |  | | | | |
| 41 | Yan *et al.*, 2011 | Talairach | 28 | 31.7 | -3.2 | 32.7 | 10.66 | | 2 > 0 back | | | | | Sea level | | | | |
|  |  |  |  | 28.1 | 59.3 | 43.6 | 9.94 | |  | | | | |  | | | | |
|  |  |  |  | -33.6 | 58.7 | 45.8 | 9.59 | |  | | | | |  | | | | |
|  |  |  |  | -0.7 | 52.4 | 25.3 | -8.49 | |  | | | | |  | | | | |
|  |  |  |  | 0.9 | -1.2 | 54.6 | 9.12 | |  | | | | |  | | | | |
|  |  |  |  | -30.6 | -16.6 | 14.1 | 9.88 | |  | | | | |  | | | | |
| 42 | Yoo *et al.,* 2004 | Talairach | 12 | 34 | 51 | 11 | 6.81 | | 2 > 1 back | | | | |  | | | | |
|  |  |  |  | -38 | 45 | 13 | 3.89 | |  | | | | |  | | | | |
|  |  |  |  | 55 | 15 | 32 | 3.77 | |  | | | | |  | | | | |
|  |  |  |  | -53 | 8 | 40 | 3.74 | |  | | | | |  | | | | |
|  |  |  |  | 57 | -48 | 2 | 3.48 | |  | | | | |  | | | | |
|  |  |  |  | 51 | -29 | -7 | 3.74 | |  | | | | |  | | | | |
|  |  |  |  | -44 | -58 | -4 | 4.18 | |  | | | | |  | | | | |
|  |  |  |  | 34 | 26 | 12 | 3.41 | |  | | | | |  | | | | |
|  |  |  |  | -38 | 21 | -3 | 3.02 | |  | | | | |  | | | | |
|  |  |  |  | -2 | -4 | 68 | 4.27 | |  | | | | |  | | | | |
|  |  |  |  | -46 | -19 | 49 | 3.50 | |  | | | | |  | | | | |
|  |  |  |  | -4 | 19 | 40 | 3.52 | |  | | | | |  | | | | |
|  |  |  |  | 34 | -46 | 45 | 5.81 | |  | | | | |  | | | | |
|  |  |  |  | -36 | -48 | 48 | 3.87 | |  | | | | |  | | | | |
|  |  |  |  | 6 | -76 | -10 | 4.44 | |  | | | | |  | | | | |
|  |  |  |  | -32 | -65 | -17 | 4.27 | |  | | | | |  | | | | |

**Supplementary Table 2**. Coordinates used for the load-effect meta-analysis

Abbreviations: N: sample size; =: same contrast used as in the main meta-analysis

|  | Study | Coordinate system | | N | x | y | z | peak (t) | Contrast |
| --- | --- | --- | --- | --- | --- | --- | --- | --- | --- |
| 1 | Altamura *et al.*, 2007 | = = | | | = | = | = | = | Load |
| 2 | Bunge *et al.*, 2001 | = | = | | = | = | = | = |  |
| 3 | Cairo *et al.*,2004 | Talairach | 18 | | -8 | 2 | 56 | 8.33 | Encoding |
|  |  |  |  | | -44 | -6 | 44 | 10.10 |  |
|  |  |  |  | | 40 | -2 | 48 | 8.79 |  |
|  |  |  |  | | -52 | 8 | 14 | 6.84 |  |
|  |  |  |  | | 52 | 8 | 18 | 7.08 |  |
|  |  |  |  | | 36 | 44 | 24 | 7.36 |  |
|  |  |  |  | | -28 | -48 | 42 | 8.30 |  |
|  |  |  |  | | 40 | -36 | 42 | 11.43 |  |
|  |  |  |  | | -24 | -64 | 36 | 13.32 |  |
|  |  |  |  | | 8 | -56 | 50 | 8.93 |  |
|  |  |  |  | | 16 | -68 | 52 | 7.86 |  |
|  |  |  |  | | -48 | -62 | 14 | 11.34 |  |
|  |  |  |  | | 48 | -54 | -8 | 7.74 |  |
|  |  |  |  | | -16 | -88 | 26 | 18.82 |  |
|  |  |  |  | | 16 | 96 | -4 | 18.00 |  |
|  |  |  |  | | -24 | 8 | 0 | 8.59 |  |
|  |  |  |  | | 16 | 16 | -4 | 11.62 |  |
|  |  |  |  | | -12 | 20 | -8 | 10.51 |  |
|  |  |  |  | | 12 | 16 | -4 | 9.82 |  |
|  |  |  |  | | -12 | 0 | 0 | 7.71 |  |
|  |  |  |  | | 16 | 0 | 4 | 7.83 |  |
|  |  |  |  | | -16 | -16 | -6 | 10.72 |  |
|  |  |  |  | | 12 | -8 | 12 | 8.62 |  |
|  |  |  |  | | -12 | 12 | -8 | 9.86 |  |
|  |  |  |  | | 12 | -16 | -2 | 10.38 |  |
|  |  |  |  | | -36 | -64 | 18 | 10.94 |  |
|  |  |  |  | | 32 | -60 | 18 | 8.36 |  |
|  |  |  |  | | 0 | 28 | 36 | 12.88 | Recall |
|  |  |  |  | | 40 | 32 | 24 | 9.74 |  |
|  |  |  |  | | 32 | 52 | 16 | 7.33 |  |
|  |  |  |  | | -4 | -78 | 16 | 8.69 |  |
|  |  |  |  | | 44 | -64 | 28 | 8.62 |  |
|  |  |  |  | | 36 | -70 | 20 | 7.27 |  |
| 4 | Caseras *et al.,* 2006 | = | = | | = | = | = | = |  |
| 5 | Chen and Desmond, 2005a | = | = | | = | = | = | = |  |
| 6 | Chen and Desmond, 2005b | = | = | | = | = | = | = |  |
| 7 | Desmond *et al.,* 2003 | = | = | | = | = | = | = |  |
| 8 | Johnson *et al.,* 2006 | = | = | | = | = | = | = |  |
| 9 | Kirschen *et al.*, 2010 | = | = | | = | = | = | = |  |
| 10 | Knops *et al.,*2006 | = | = | | = | = | = | = |  |
| 11 | Lythe *et al.*, 2012 | = | = | | = | = | = | = |  |
| 12 | Ragland *et al.*, 2002 | Talairach | 11 | | 28 | 26 | 8 | 8.75 | 2 > 1 |
|  |  |  |  | | -48 | 14 | 8 | 7.81 |  |
|  |  |  |  | | 36 | 46 | 20 | 8.74 |  |
|  |  |  |  | | -36 | 26 | 24 | 9.25 |  |
|  |  |  |  | | -44 | 6 | 36 | 8.26 |  |
|  |  |  |  | | 40 | 6 | 40 | 9.80 |  |
|  |  |  |  | | -12 | -10 | 16 | 7.39 |  |
|  |  |  |  | | 4 | 22 | 36 | 7.81 |  |
|  |  |  |  | | 44 | -46 | 40 | 10.39 |  |
|  |  |  |  | | -24 | -58 | 40 | 9.81 |  |
| 13 | Schloesser *et al.*, 2008 | = | = | | = | = | = | = |  |
| 14 | Veltman *et al.*, 2003 | = | = | | = | = | = | = |  |
| 15 | Wolf *et al.,* 2006 | = | = | | = | = | = | = |  |
| 16 | Yoo *et al.,* 2004 | = | = | | = | = | = | = |  |
|  |  |  |  | |  |  |  |  |  |

## Supplementary Figures


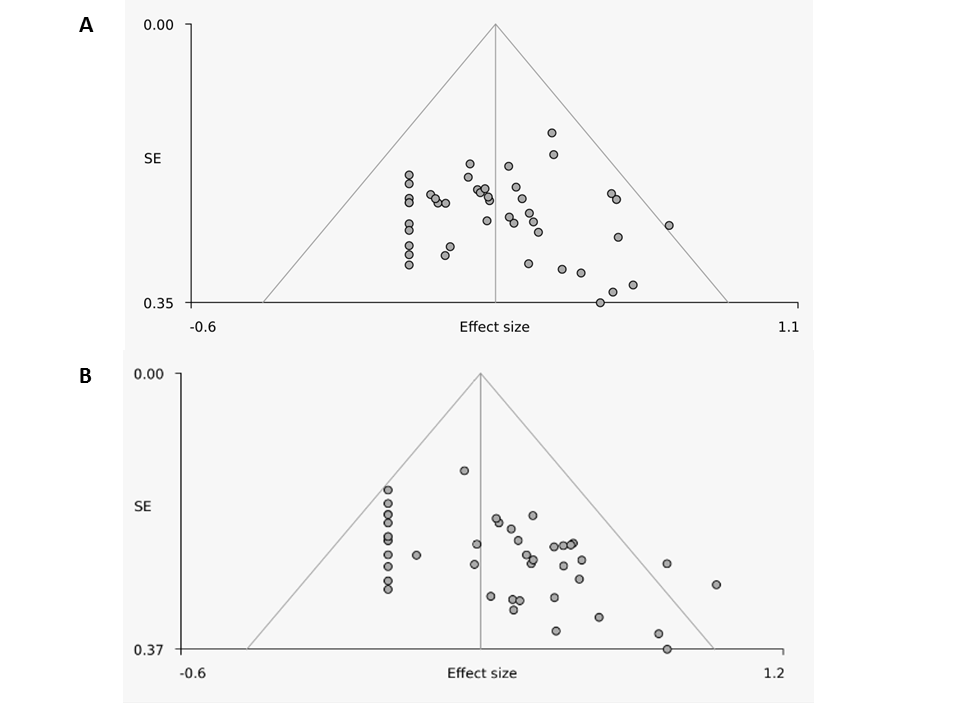


**Supplementary Figure 1.** Examples of symmetric and asymmetric funnel plots. **A:** MNI coordinates: 50,25,2 (right inferior gyrus, opercular part); p=0.732, **B:** MNI coordinates: 40,-58, 44 (right angular gyrus); p=0.001, SE: standard error.
